# A numerical approach to overcome the very-low Reynolds number limitation of the artificial compressibility for incompressible flows

**DOI:** 10.1016/j.heliyon.2024.e39587

**Published:** 2024-10-21

**Authors:** Nikos Monokrousos, László Könözsy, Vassilios Pachidis, Ernesto Sozio, Federico Rossi

**Affiliations:** a Cranfield University, College Road, Cranfield, Bedfordshire, MK43 0AL, United Kingdom; b Pangea Aerospace, Avinguda Número 1, 20 08040 Barcelona, Spain

**Keywords:** Artificial compressibility, Pseudo-compressibility, Incompressible flows, Low Reynolds number flows, Oseen flow, Internal flows, Higher-order pressure boundary conditions

## Abstract

We propose a numerical approach to solve a long-standing challenge which is the applicability of the artificial compressibility (AC) formulation for solving the incompressible Navier—Stokes equations at very-low Reynolds numbers. A wide range of engineering applications involves very-low Reynolds number flows in Micro-ElectroMechanical Systems (MEMS) and in the fields of chemical-, agricultural- and biomedical engineering. It is known that the already existing numerical methods using the AC approach fail to provide physically correct results at very-low Reynolds numbers (*Re* ≤ 1). To overcome the limitation of the AC method for these engineering applications, we propose a higher-order Neumann-type pressure outflow boundary condition treatment along with their up to fourth-order numerical approximations. We found that the numerical treatment of the pressure at the outlet boundary plays the main role in overcoming the limitation of the AC method at very-low Reynolds numbers (*Re* << 1). Therefore, we provide numerical evidence on the accuracy of the AC method beyond its previously reported limitations, e.g., the low Reynolds number Oseen flow (*Re* << 1) is first presented in this work. A third-order explicit total-variation diminishing (TVD) Runge–Kutta scheme has been employed with standard finite difference spatial discretisation schemes for improving the accuracy of the numerical solution. For modelling strongly viscous flows, the Reynolds number ranges from $10^{-1}$ to $10^{-4}$. Overall, we found that the accuracy limitation of the AC method below *Re* < 1 can be overcome with an accurate numerical treatment of the outlet pressure boundary condition instead of using high-order schemes in the governing equations. For the investigated Reynolds number range ($10^{-1}$ ≤ Re ≤ $10^{-4}$), the obtained results show that the relative errors were smaller than 1% for the numerical simulations performed on the configurations of both the two- and three-dimensional, straight microfluidic channels. The imposition of high-order derivative Neumann-type pressure outflow boundary conditions reduced the maximum relative errors of the numerical solutions from 85% and 95% to below than 1% at the outlet section of the two- and three-dimensional, straight microfluidic channel flows, respectively. Taking the advantage of the numerical approach proposed here, two- and three-dimensional benchmark problems employed in the current investigation in comparison with analytical solutions available in the literature, clearly demonstrate that the artificial compressibility can be used beyond its previously known constraints for very-low Reynolds number incompressible flows.

## Highlights

• Overcoming limitations of the artificial compressibility (AC) at very-low Re number flows.
• Limitations of the AC method found to originate from the pressure boundary condition treatment.
• High-order pressure outlet boundary conditions up to fourth-order accuracy are proposed.
• 2D and 3D benchmark problems compared against analytical solutions at Re << 1.
• Oseen problem solution with the AC method is first time presented at Re << 1.

## Nomenclature

**Abbreviations**
**AC**: Artificial Compressibility
**BVP**: Boundary Value Problem
**CB**: Characteristics-Based
**CFL**: Courant–Friedrichs–Lewy
**FDM**: Finite Difference Method
**FS-PP**: Fractional-Step Pressure-Projection
**FSAC-PP**: Fractional-Step Artificial Compressibility with Pressure-Projection
**FVM**: Finite Volume Method
**GCI**: Grid Convergence Index
**IVP**: Initial Value Problem
**MEMS**: Micro-ElectroMechanical Systems
**PVC**: Pressure-Velocity Coupling
**TVD**: Total-Variation Diminishing

**Symbols**
u: Velocity-field vector
CFLinv: Inviscid CFL number
CFLvis: Viscous CFL number
Dh: Hydraulic diameter
$e_{a}^{21}$: Approximate relative error
$e_{ext}^{21}$: Extrapolated relative error
$GCI_{fine}^{21}$: Fine-grid convergence index
Mfluid: Molar mass of the fluid substance
$N_{1}$: Total number of cells for fine mesh
$N_{2}$: Total number of cells for intermediate mesh
$N_{3}$: Total number of cells for coarse mesh
NA: Avogadro constant
$r_{21}$: Intermediate to fine grid refinement factor
$r_{32}$: Coarse to intermediate grid refinement factor
*dx*: Grid-spacing in direction *x*
*dy*: Grid-spacing in direction *y*
*dz*: Grid-spacing in direction *z*
D: Denominator of rational coefficients
H: Height of the channel
i: Spatial grid index in direction *x*
j: Spatial grid index iin direction *y*
k: Spatial grid index iin direction *z*
L: Length of the channel
n: Previous pseudo-time level
N: Numerator of rational coefficients
p: Hydrodynamic pressure
Q: Volumetric flow rate
Re: Reynolds number
*RHSP*: Right-hand side of perturbed equation
*RHSU*: Right-hand side of *x-momentum equation*
*RHSV*: Right-hand side of *y-momentum equation*
*RHSW*: Right-hand side of *z-momentum equation*
U: Uniform mean flow velocity
u: Velocity component in direction *x*
v: Velocity component in direction *y*
W: Width of the channel
w: Velocity component in direction *z*

**Greek Symbols**
*α*: Order of approximation accuracy
β: Pseudo-compressibility parameter
Δτ: Pseudo-time step
$\Delta \tau_{inv}$: Inviscid pseudo-time step
$\Delta \tau_{vis}$: Inviscid pseudo-time step
$\Delta_{p}$: Pressure difference between inlet and outlet
ϵ: Designated convergence tolerance
γ: Safety factor
κ: Index of summation
λ: Index of summation
$\lambda_{fluid}$: Average interatomic spacing
μ: Dynamic viscosity of the fluid
ν: Kinematic viscosity of the fluid
ρ: Density of the fluid
τ: Pseudo-time
$\phi_{1}$: Solution on fine mesh
$\phi_{2}$: Solution onr intermediate mesh
$\phi_{3}$: Solution on coarse mesh
$\phi_{ext}^{21}$: Extrapolated values

## 1 Introduction

A mathematical modelling approach within the context of the Artificial Compressibility (AC) pressure-velocity coupling (PVC) method is presented for the solution of incompressible stationary Navier-Stokes and Oseen equations for hydrodynamic channel flows at very-low Reynolds numbers (*Re* << 1). The numerical approach proposed here shows how to overcome the computational of the AC method for viscous-dominant flows through an appropriate treatment of the outlet pressure boundary condition, by imposing higher-order derivative Neumann-type conditions.

Very-low Reynolds number flows are of great importance for a wide variety of industrial applications and engineering fields. The dimensionless Reynolds number accounts for the ratio of the inertia to the viscous forces applied in the fluid flow and the overall fluidic system [1], thus low- and very-low Reynolds number flows are characterised by the relative predominance of the latter over the former. The utilisation of dimensionless numbers indicates the flow effects that need to be taken into consideration [2]. Very-low Reynolds number flows are exhibiting strongly viscous behaviour, an expression introduced by Shapiro [3] in 1964. These types of fluid flows are expected to be found in micro- and nanofluidic systems requiring the description of the fluid flow motion using either the continuum or particle-based approaches [4]. In fact, the vast majority of microfluidic applications are characterised by Reynolds numbers of the order of magnitude below one (*Re* << 1), as a consequence of the small characteristic dimensions of the microfluidic channel configurations [5]. A number of industrial and engineering applications involve low and very-low Reynolds number internal flows, such as microfluidic flows with in-situ membrane formations [6], [7] and flows in Micro-ElectroMechanical Systems (MEMS) [8], [9], [10], flows in tube coils and helical pipes [11], [12], and Darcian flows through porous media and constricted tubes in the fields of chemical- and agricultural-engineering [7], [13], [14], [15]. Over the last few years, there is an increased interest in bioengineering applications governed by viscous flows. Numerical and experimental biomedical studies are available in the literature for low- and very-low Reynolds number flows, e.g., wall-bounded blood flows [16], [17], [18] and flows in surgical procedures and biomedical microdevices [19], [20], [21], [22].

The mathematical description of a fluid flow incorporates a numerical approach to the governing equations, which is a technique for the resolution of the variables of interest along with a solution algorithm for computer code implementation purposes. In the present work, the Artificial Compressibility (AC) pressure-velocity coupling formulation of Chorin [23] is employed for solving the incompressible Navier-Stokes equations. The AC method incorporates a pseudo-time derivative of the pressure into the continuity equation of incompressible flows, yielding a perturbed continuity equation and allowing for the prediction of the evolutionary behaviour of the pressure-field. For steady-state flows, the time-stepping strategy is implemented with respect to pseudo-time. Once a steady-state solution is achieved, the principal stationary form of the Navier-Stokes equations is retrieved [24]. The introduction of an AC or pseudo-compressibility parameter *β* in the perturbed continuity equation, transforms the system of governing equations into a set of hyperbolic-type equations [25]. The parameter *β* behaves similarly to a convergence parameter and allows the governing equations to march in pseudo-time whilst satisfying the incompressibility (divergence-free) constraint of the velocity-field [26]. The AC parameter has no particular physical meaning with respect to the propagation of the pressure waves, and is responsible for the propagation of the numerical pressure waves due to its presence in the perturbed continuity equation. In the context of the pseudo-compressibility method, the solution of a pressure-Poisson equation is not required and therefore, the numerical waves behave as the medium for the pressure distribution [27].

Kwak et al. [27] provided a comprehensive review on the development of incompressible fluid flow solvers since 1930s. In their review article [27], the “artificial compressibility” or “pseudo-compressibility” method of Chorin [23] was mentioned in the class of “density-based” type solvers. At this point, it is important to distinguish between pressure- and density-based mathematical formulations of the continuity equation as a clarification. It is known that the mass conservation equation of compressible fluid flows contains the density variation in which case the real speed of sound can be taken into account. For incompressible flows, the density variable is constant and a pressure perturbation term was introduced by Chorin [23] in the continuity equation, which is a numerical perturbation technique to obtain a pseudo-transient pressure-velocity coupling closure model for low Mach and low Reynolds number flows strictly with constant density. In other words, due to the presence of the pressure perturbation term, the incompressibility (divergence-free) constraint, or the continuity equation, of incompressible flows is satisfied iteratively instead of immediately in the numerical solution procedure. Therefore, the pseudo-compressibility method of Chorin [23] for solving the incompressible Navier–Stokes equations turns out to be a pressure-based solver, since the pressure perturbation term iteratively vanishes from the perturbed continuity equation through a pseudo-transient numerical solution. For example, when characteristics-based (CB) schemes are employed for the discretisation of the non-linear convective/advective terms in the context of the Chorin's method [28], [29], [30], [31], the definition of the artificial speed of sound can be introduced and taken into account as a numerical parameter where the density still remains constant, i.e., the fluid flow remains again inherently incompressible. The pseudo-compressibility method of Chorin [23] is not a compressible flow solver at low Mach numbers. In the context of incompressible fluid flow solvers and pressure-velocity coupling methods, the name of “artificial compressibility” or “pseudo-compressibility” could be a misleading expression, because the incompressible, pseudo-transient pressure-velocity coupling formulation of Chorin [23] has inherently ignores any real compressibility effect, i.e., density variation in the fluid flow-field. Strictly speaking, the pseudo-transient pressure-velocity coupling algorithm of Chorin [23] is solely a mathematical modelling approach to close the system of governing equations for solving the incompressible Navier–Stokes equations as a type of pressure perturbation added to the divergence-free continuity equation of incompressible flows. Therefore, instead of using the name of “artificial compressibility” or “pseudo-compressibility” method for the incompressible flow solver of Chorin [23], the expression of “pseudo-transient pressure-velocity coupling approach” or “pseudo-transient perturbed continuity equation formulation” for solving incompressible fluid flow problems would rather be recommended by the authors, to avoid any misunderstanding in terms of the numerical modelling nature of constant density flows.

The stiffness of the perturbed continuity equation in conjunction with the different numerical solution approaches for very-low Reynolds number incompressible flow was highlighted by different authors. Peyret and Taylor [32] investigated the mesh size of the computational domain and its relationship with the efficiency of the AC method in the 1980s. Their numerical experiments were valid for Reynolds numbers in the range between 1 and 10^3^ using a finite difference method. Furthermore, Peyret and Taylor [32] pointed out the disadvantageous properties of the standard finite difference discretisation schemes in the framework of the AC method, therefore, applied mathematicians and computational scientists were encouraged to explore other discretisation approaches for solving the governing equations of the pseudo-compressibility formulation in that time. Drikakis et al. [28] proposed a single directional characteristics-based (CB) treatment for the non-linear convective flux terms in the framework of Godunov-type methods for solving the incompressible Navier-Stokes equations with the AC method of Chorin [23]. In their work, the importance of the AC or pseudo-compressibility parameter *β* was highlighted for the determination of the local pseudo-time step at very-low Reynolds numbers. An inappropriate selection of the AC parameter *β* can result in slow numerical convergence rates of the numerical solution and lead to a non-physical solution of the investigated physical problem for Re ≪1. Up-to-date, there is no general mathematical relationship between the AC parameter *β* and the Reynolds number. Many attempts have been made for the mathematical formulation of the correlation between the AC parameter *β* and the dimensionless Reynolds number. In the work of Chorin [23], the AC parameter *β* was introduced as a convergence parameter. Chorin investigated the numerical solution of an incompressible, viscous flow in a segment of a channel and was the first to report that the case-dependent, optimal value of the pseudo-compressibility parameter, determined through numerical experiments, decreases with the increment of the investigated flow Reynolds number. Rogers and Kwak [33] examined the two-dimensional benchmark test cases of the square driven cavity, backward-facing step flow and a flow around a circular cylinder. Using the pseudo-compressibility method for the numerical solution of the two-dimensional Navier–Stokes equations, they were able to exhibit the sensitivity of the solution convergence rate to the value of the AC parameter *β*. Furthermore, after performing numerical convergence tests for the determination of the pseudo-compressibility parameter, they also concluded in reducing the parameter value while investigating flows of increasing Reynolds number. In a recent study from Könözsy and Drikakis [31] on a two-dimensional, straight microfluidic channel, they accordingly proceeded with numerical experiments for the estimation of the pseudo-compressibility parameter, proving the disproportionality between the Reynolds number of the microfluidic channel flow and the AC parameter *β*. Despite the fact that currently numerical investigations indicate disproportionality between the parameter *β* and the Reynolds number of the examined flow [23], [31], [33], it is still unknown how the computational limitations originating from the employment of the AC method in the context of the standard finite difference method (FDM) in very-low Reynolds number flows, where the pseudo-compressibility parameter is characterised by large values, can be overcome. The numerical approach presented in the current work aims to bridge this research gap and extend the computational capabilities of the pseudo-compressibility method. A comprehensive analysis on the AC formulation of Chorin [23] can be found in the textbook of Temam [34].

The CB numerical treatment of the governing equations of the pseudo-compressibility method obtained an impetus among applied mathematicians and computational scientists from the 1990s until nowadays. The standard finite difference discretisation of the AC formulation of Chorin [23] was abandoned for a long time. The focus on the CB schemes in conjunction with the AC method became a leading research area for the development of pseudo-transient formulations for solving incompressible fluid flow problems. In the work of Shapiro and Drikakis [29], [30], the authors further improved the single-directional CB treatment of the non-linear convective terms by investigating the accuracy and efficiency of the AC pressure-velocity coupling method for the numerical solution of different constant and variable density, stationary and unsteady laminar flow problems, when imposing the divergence-free constraint of the velocity-field. The importance for the selection of an appropriate numerical value for the pseudo-compressibility parameter *β* to control the rate of numerical convergence was discussed in both studies. The computational inefficiency of the AC method in conjunction with CB schemes for very-low Reynolds number flows was also reported in different research papers and studies. Zhao and Zhang [35] made an attempt to use a high-order CB upwind Finite Volume Method (FVM) approach and found that the convergence rate of their method was much slower at low Reynolds numbers compared to moderate Reynolds numbers for a two-dimensional backward-facing step benchmark problem. Zamzamian and Hashemi [36] carried out a development and investigation on multi-directional (multi-dimensional) CB schemes in particular attention to the treatment of the solid boundaries for predicting incompressible fluid flows. Könözsy and Drikakis [31] proposed a unified Fractional-Step Artificial Compressibility with Pressure-Projection (FSAC-PP) numerical solution approach to simulate very-low Reynolds number incompressible flows up to *Re* = $10^{-4}$ for two-dimensional microfluidic channel flows. Their unified pressure-velocity coupling approach was developed in the context of single-directional CB discretisation of the pseudo-transient governing equations for the incompressible Navier–Stokes equations instead of using standard finite difference approximations in discretisation of the governing equations. They successfully identified and reported the computational stiffness of the AC method of Chorin [23] for very-low Reynolds number flows. Teschner et al. [37] focused on the further development of the FSAC-PP approach in the context of multi-directional CB schemes to improve the accuracy and efficiency of the numerical solution of the incompressible Navier–Stokes equations for moderate Reynolds number flows. Nithiarasu and Liu [38] also employed the pseudo-compressibility formulation of Chorin [23] using CB schemes for the numerical solution of steady and unsteady, incompressible turbulent flows including a flow past a circular cylinder and a model of the upper human airway at moderate Reynolds number, exhibiting the appropriateness of their numerical formulation. Therefore, the demand is still there to explore numerical solution strategies to overcome the limitations of the hyperbolic-type AC method of Chorin [23] for very-low Reynolds number incompressible flows in the framework of low- and high-order finite difference approximation methods. It is important to note that the application of the pseudo-compressibility method of Chorin [23] in the discretisation framework of the standard finite difference method (FDM) has received very little attention over the past thirty years within the scientific research community.

Ekaterinaris [39] presented the numerical solution of the incompressible Navier–Stokes equations using the pseudo-compressibility method and high-order accurate, central finite difference approximations for the investigation of the accuracy and computational efficiency of the AC method for stationary benchmark flow test cases, including flows in a pipe, channel and boundary layer formation behind a flat plate. In that work, the moderate Reynolds number flows were examined for each case. Rogers et al. [40] employed a standard finite difference method for the investigation of the accuracy and efficiency of the pseudo-compressibility method for solving the incompressible Navier–Stokes equations. Their numerical results were compared to experimental data for a two-dimensional backward-facing step flow and a flow over an impulsively started circular cylinder with the use of the implicit approximate factorization scheme. A thorough discussion was presented for the determination of the pseudo-compressibility parameter *β* and the influence of its selection on the numerical convergence. In the context of standard finite difference (FDM) discretisation techniques, these studies underlined the statements made by Peyret and Taylor [32], Karniadakis et al. [5] and Drikakis and Rider [25] regarding the limitation of the pseudo-compressibility method of Chorin [23] at very-low Reynolds number flows (*Re* << 1). In the present study, the main contribution is to provide a numerical framework in the context of standard finite difference method on how to overcome the limitation of the pseudo-compressibility method of Chorin [23] at *Re* < 1. Therefore, the numerical investigation of the low Reynolds number Oseen flow (*Re* << 1) has been presented for the first time in this work in conjunction with the pseudo-compressibility method.

As shown in the numerical approach presented in this work, the selection of the mathematical formulation of the boundary condition for the pressure-field at the outlet section plays a key role to overcome the computational limitation of the pseudo-compressibility approach at very-low Reynolds number flows (*Re* << 1). In the primitive variables formulation of the governing equations, physically inappropriate boundary conditions for the pressure-field have significant impact on the accuracy of the standard finite difference solution method. The importance of the specification of the boundary condition for the pressure in the research field of incompressible fluid flows was highlighted by Gresho and Sani [41] and Johnston and Liu [42], among others. In the former study, Gresho and Sani [41] brought attention to the importance of imposing appropriate boundary conditions for the pressure variable, in the context of the solution of a Poisson equation for benchmark test cases. Johnston and Liu [42] additionally employed Neumann-type boundary conditions for the solution of the associated pressure-Poisson equation required to demonstrate their development of a second-order numerical scheme. For the solid boundaries, the pressure gradient perpendicular to the wall pointing outwards is suggested to be set equal to zero. For incompressible flows, pressure performs as a mathematical constraint for the velocity-field to sustain its divergence-free characteristics. Numerical investigations for spectral approximations [43] were performed by Moin and Kim [44], Kleiser and Schumann [45] and Deville et al. [46] on the influence of different pressure variable treatments in the context of pressure-projection methods, in which case a pressure-Poisson equation has to be solved. Initially, Moin and Kim [44] suggested the direct solution of the continuity equation instead of the pressure-Poisson equation for viscous, incompressible flows, to avoid potential numerical issues arising from the imposition of appropriate boundary conditions for the Poisson equation. In their suggested pseudospectral formulation, the necessity for the employment of pressure boundary conditions on the wall boundaries of the investigated geometric configurations was ultimately eliminated. The spectral method utilised by Kleiser and Schumann [45] for the numerical solution of Poiseuille and Couette flows was based on an influence matrix technique for the generation of pressure boundary conditions that fulfill the kinematic constraint of the continuity equation in the Navier–Stokes governing equations. Deville et al. [46] also investigated the influence of the pressure variable treatment on the numerical solution of a Stokes flow on a slab geometric configuration, in the context of spatial spectral approximations. In the context of the pseudo-compressibility method of Chorin [23], special treatment is required for the pressure boundary conditions due to the absence of a pressure-Poisson equation. For using characteristics-based discretisation schemes, Drikakis and Rider [25] proposed the determination of the inflow and outflow pressure boundary conditions based on the characteristic waves propagating throughout the computational domain. The finite volume method (FVM) was employed by Georgantopoulou and Tsangaris [47] in conjunction with the pseudo-compressibility method for the numerical solution of the two-dimensional lid cavity and a stenosed tube in curvilinear domains at *Re* = 100. For modelling incompressible turbulent flows, Haskew and Sharif [48] employed standard finite difference approximations [49] along with the pseudo-compressibility pressure-velocity coupling formulation providing a very accurate estimation for the velocity distribution and the pressure loses in vaned pipe bends. Note that standard finite difference schemes were as a matter of fact employed in the original work of Chorin [23] introducing the pseudo-compressibility method, which could still inspire research activities in this scientific area.

By exceeding the computational limitation of the pseudo-compressibility method at very-low Reynolds number incompressible flows (*Re* << 1), its hyperbolic-type formulation can advantageously be used in conjunction with high-fidelity and high-order computational techniques. The simplicity of the implementation of the pseudo-compressibility method of Chorin [23] is another advantageous feature of this method which can be used for a wide range of engineering problems including very-low Reynolds number incompressible flows presented here. The method is becoming increasingly more popular for various practical applications involving both compressible and incompressible flows in a range of Reynolds numbers. For instance, the overall performance of Chorin's [23] pseudo-compressibility method was successfully investigated in conjunction with quasi-Newton methods for fully-enclosed, incompressible fluid-structure interactions [50] or for smoothed particle hydrodynamics applications requiring the characterisation of the fluid-structure interactions for incompressible flows [51]. The solution of the variable-density governing equations based on a Godunov-type scheme for applications involving free-surface simulations was also examined [52]. Mandal et al. [53] further investigated unsteady, incompressible channel flows over moving boundaries in two-dimensional planes within the context of the pseudo-compressibility method of Chorin [23]. In their work, they proposed a novel upwind method based on an extension of the Harten Lax and van Leer with Contact (HLLC) method coupled with the Artificial Compressibility (AC) method and the Arbitrary Lagrangian Eulerian (ALE) formulation for the dual time-stepping technique to examine moderate Reynolds number flows. When employed for the numerical solution of the pseudo-compressibility formulation, the proposed HLLC-AC upwind method appeared to perform in very good agreement in terms of capturing the flow-field characteristics, in comparison with experimental and computational results available in the literature. Following the development of a novel HLLC-AC upwind method [53], Mandal and Sonawane [54] investigated the computational capabilities of the pseudo-compressibility method of Chorin [23] in different geometric configurations and flow conditions. For instance, based on the developed upwind method in the context of the pseudo-compressibility formulation of Chorin [23], they reported the numerical simulation solutions of a liquid silicon flow in a differentially-heated, orthogonal-rotating, square cavity for both an inertial and a non-inertial reference frame of the governing equations [54] and moderator flows inside calandria of the typical CANDU-6 reactor [55], with rather promising results. The HLLC-AC upwind method [53] was also extended to account for Solution Dependent Weighted Least Squares (SDWLS) gradients calculations to achieve higher-order accuracy over unstructured meshes. The updated SDWLS upwind method was tested for the case of unsteady channel flows with a moving indentation [56] and for fluid-interaction problems involving heat transfer phenomena such as an annulus heat exchanger characterised by helical flow passages [57], flow-induced vibrations originating from the cyclic lift forces over a cooled circular cylinder [58] and forced heat transfer from the circular cylinder forced to oscillate in an elliptical path in an incompressible fluid flow for unsteady, laminar flows of Reynolds number equal to 100, 150 and 200 [59], with the results exhibiting very good agreement with experimental and computational results available in the literature. Therefore, the Artificial Compressibility (AC) method of Chorin [23] is still highly influential for the development of numerical schemes and formulations and the solution of different geometric configurations and flow conditions. From this perspective, the suggestion of novel numerical approaches able to extend the computational capabilities of the pseudo-compressibility method is of uttermost importance. As discussed, the diverse landscape of the existing literature exhibits a plethora of research gaps in the field of numerical methods for the solution of fluid flow governing equations. For instance, within the context of Magnetohydrodynamics (MHD), numerous investigations are still being performed for the characterisation of biomagnetic fluid flows under the influence of magnetic fields varying with respect to space. Similar to the numerical investigation reported in the present work, the standard finite difference method has been widely employed for the numerical solution of fluid flow governing equations accounting for magnetic phenomena through the inclusion of magnetisation and Lorentz forces. The influence from the presence of a magnetic field on the flow-field development was exhibited for a stationary, two-dimensional, incompressible, isothermal channel flow in the laminar regime [60] and in the case of a stretching cylinder for non-isothermal blood flow through a cylindrical tube [61]. A Galerkin weighted residual method was also employed for the finite element analysis of a non-isothermal, two-dimensional, laminar flow of an incompressible, Newtonian fluid in a straight, channel of rectangular cross-section, characterised by *Re* = 250, exhibiting very good agreement with data available in the literature [62]. In the cases where a transverse magnetic field is present, the governing equations for the flow-field development and heat transfer characterisation may need to transform into a system of coupled non-linear ordinary differential equations (ODEs) using the similarity conversion approach [63]. Therefore, the necessity for numerical approaches for the pressure-velocity coupling of fluid flow governing equations is still apparent, regardless of the advancements in the field of numerical methods. The numerical approach proposed in this work is capable of overcoming the limitations of the pseudo-compressibility method for very-low Reynolds number, steady-state, incompressible flows by imposing higher-order Neumann-type outflow boundary conditions with their up to fourth-order numerical treatment. Two- and three-dimensional flow problems in channels will be considered in which cases the accuracy and efficiency of the pseudo-compressibility approach was demonstrated beyond its previously reported limitations in the literature [5], [25], [32]. The presented work is in alignment with the research gaps identified in the literature for the numerical solution of viscous flows of high engineering and scientific relevance.

The structure of the current work is as follows. In Section 2, a numerical solution approach is presented to overcome the limitation of the pseudo-compressibility pressure-velocity coupling method of Chorin [23] for solving the incompressible Navier–Stokes equations at very-low Reynolds numbers (*Re* << 1). To achieve this goal, higher-order Neumann-type pressure outlet boundary conditions along with their up to fourth-order numerical treatment are proposed. For the sake of simplicity, cartesian coordinates have been used. The details of the explicit standard finite difference discretisation approach of the numerical solver are described. In Section 3, computational results in comparison with analytical solutions obtained through the implementation of the proposed numerical approach are discussed for very-low Reynolds number flows (*Re* << 1) in addition to the analytical solutions and the mesh sensitivity study for each validation benchmark problem. Three benchmark problems describing incompressible, steady-state flows have been selected for validation purposes as follows: a) laminar flows in a straight microfluidic channel (*Re* << 1), b) two-dimensional flows in a channel governed by the Oseen equations (*Re* << 1), and c) three-dimensional flows in a rectangular channel governed by the incompressible Navier–Stokes equations. The numerical experiments for the determination of the pseudo-compressibility parameter *β* are also exhibited. In Section 4, the conclusions of the numerical approach to overcome the limitation of the AC method proposed in this work are briefly summarised.

## 2 Numerical approach to overcome the limitation of the pseudo-compressibility method at *Re* << 1

To address the solution of a long-standing challenge in the field of applied mathematical modelling—which is on how to overcome the limitation of the pseudo-compressibility method of Chorin [23] for very-low Reynolds number incompressible flows (*Re* << 1) in the context of standard finite difference methods—, a numerical approach with higher-order Neumann-type pressure outflow boundary conditions along with their up to fourth-order numerical treatment are proposed in this Section. Details on the implementation of the numerical solver and schemes employed for the temporal and spatial discretisation of the governing equations of steady-state, incompressible flows are presented. The motivation for the research is relying on the numerical observation that the mathematical treatment of the pressure at the outlet boundary indeed plays the main role in overcoming the limitation of the pseudo-compressibility method of Chorin [23] using the standard finite difference method (FDM) at very-low Reynolds numbers (*Re* << 1). In Section 3, numerical results compared to analytical solutions for benchmark problems will be provided in detail.

### 2.1 Governing equations of stationary, incompressible flows

The system of the governing equations for the description of steady-state, incompressible flows consists of the mass conservation (continuity) equation and the Navier-Stokes momentum equations. Assuming that no external forces are acting on the fluid body, the dimensional governing flow equations are written in vector form as

(1) ∇⋅u=0,

(2) $$\left(u\cdot \nabla \right)u=-\frac{1}{\varrho }\nabla p+\nu \nabla^{2}u,$$

where **u** represents the velocity-field, *ρ* the density of the fluid, *p* the hydrodynamic pressure and *ν* the kinematic viscosity of the fluid. Contrary to the momentum Eq. (2), the continuity Eq. (1) does not include the pressure variable. Hence, in the case of incompressible flows, the governing equations are fully decoupled. The hyperbolic-type Artificial Compressibility (AC) and the elliptic-type Fractional-Step Pressure-Projection (FS-PP) pressure-velocity coupling methods of Chorin [23], [64] are among the most well-known formulations for the mathematical modelling and the numerical solution of the incompressible Navier–Stokes equations [25]. Further details and numerical issues regarding stability and convergence arising from the utilisation of fractional step (or pressure-projection) methods for the solution of incompressible flows are found in the overview work of Guermond et al. [65]. The former AC pressure-velocity coupling technique constitutes the basis of the numerical approach proposed in this work for the solution of steady-state, incompressible, hydrodynamic channel flows at very-low Reynolds numbers (*Re* << 1).

### 2.2 The artificial compressibility (AC) or pseudo-compressibility method of Chorin

The Artificial Compressibility (AC) method is a pressure-velocity coupling numerical technique for the solution of the incompressible fluid flow equations introduced by Chorin [23]. The introduction of the pseudo-compressibility parameter *β* and a pseudo-time pressure derivative in the mass conservation (continuity) Eq. (1) results in a perturbed continuity equation which allows for the evolutionary determination of the pressure-field in pseudo-time iteratively. The AC formulation for the coupling of the dimensional, steady-state, incompressible Navier–Stokes governing equations consists of the perturbed continuity and the momentum equations that can be written in vector form as

(3) $$\frac{1}{\beta }\frac{\partial p}{\partial \tau }+\nabla \cdot u=0,$$

(4) $$\frac{\partial u}{\partial \tau }+\left(u\cdot \nabla \right)u=-\frac{1}{\varrho }\nabla p+\nu \nabla^{2}u,$$

where *β* is the artificial compressibility (AC) or pseudo-compressibility parameter, and *τ* is the pseudo-time. In that case, the pressure in the perturbed continuity Eq. (3) is not a function of the fluid density *ϱ*, i.e., p≠p(ϱ). The perturbed continuity and momentum Navier–Stokes system of Eqs. (3)–(4) can be expressed in scalar forms as

(5) $$\frac{1}{\beta }\frac{\partial p}{\partial \tau }+\left(\frac{\partial u}{\partial x}+\frac{\partial v}{\partial y}+\frac{\partial w}{\partial z}\right)=0,$$

(6) $$\frac{\partial u}{\partial \tau }+\left(u\frac{\partial u}{\partial x}+v\frac{\partial u}{\partial y}+w\frac{\partial u}{\partial z}\right)=-\frac{1}{\varrho }\frac{\partial p}{\partial x}+\nu \left(\frac{\partial^{2}u}{\partial x^{2}}+\frac{\partial^{2}u}{\partial y^{2}}+\frac{\partial^{2}u}{\partial z^{2}}\right),$$

(7) $$\frac{\partial v}{\partial \tau }+\left(u\frac{\partial v}{\partial x}+v\frac{\partial v}{\partial y}+w\frac{\partial v}{\partial z}\right)=-\frac{1}{\varrho }\frac{\partial p}{\partial y}+\nu \left(\frac{\partial^{2}v}{\partial x^{2}}+\frac{\partial^{2}v}{\partial y^{2}}+\frac{\partial^{2}v}{\partial z^{2}}\right),$$

(8) $$\frac{\partial w}{\partial \tau }+\left(u\frac{\partial w}{\partial x}+v\frac{\partial w}{\partial y}+w\frac{\partial w}{\partial z}\right)=-\frac{1}{\varrho }\frac{\partial p}{\partial z}+\nu \left(\frac{\partial^{2}w}{\partial x^{2}}+\frac{\partial^{2}w}{\partial y^{2}}+\frac{\partial^{2}w}{\partial z^{2}}\right),$$

where *u*, *v* and *w* are the velocity components in the *x*-, *y*- and *z*-directions, respectively. In the case of stationary, incompressible flows, time-marching is performed on pseudo-time, until a steady-state solution is obtained and the fundamental form of the Navier–Stokes equations is recovered [24]. The characteristics of the pseudo-compressibility parameter *β* and its importance for the overall performance of the numerical solution have been discussed. The determination of the parameter *β* is case-dependent; an optimal value is not available in the literature [66]. Several numerical investigations have reported the influence of the parameter on the overall performance of the numerical solver [26], [67] and the selection of extreme values resulting in the divergence [68] and instability [33] of the numerical solutions. In the presented numerical approach, the exact value of the parameter is based upon numerical experiments on the convergence rate of the obtained solutions, for different parameter values at a fixed Reynolds number.

### 2.3 Numerical implementation details

The proposed solution algorithm for the Navier–Stokes equations is presented in the dimensional formulation of primitive variables. The approach utilises the standard finite difference method (FDM) for the spatial discretisation, in conjunction with a multi-stage explicit Runge-Kutta scheme for the temporal discretisation and the explicit pseudo-time advancement of the numerical solution towards a steady-state solution. A first-order backward differencing scheme is employed for the momentum convection and the divergence of the velocity-field, a second-order centred differencing scheme for the momentum diffusion and a first-order forward differencing scheme for the pressure-field variable. Rather than the high-order spatial treatment of the terms constituting the governing equations, baseline, low-order numerical schemes are selected, to solely emphasize on the treatment of the pressure outlet boundary condition for exceeding the computational stiffness of the Artificial Compressibility (AC) method in very-low Reynolds numbers. According to Godunov's order barrier theorem [69], only up to first-order truncation error approximations for the discretisation of a system of partial differential equations allow for the preservation of its monotonic behaviour. For this reason, the employment of a first-order backward differencing scheme for the momentum convection can be beneficial for the retainment of the monotonicity of the numerical solution. In addition, low-order approximations for the discretisation of the fluid motion governing equations can result in a significant decrease of the computational time required to achieve steady-state numerical solutions for engineering applications. However, higher-order approximations can still be employed for the spatial discretisation, e.g., a fourth-order centred differencing scheme for the momentum diffusion which similarly to the second-order centred approximations has been found to be stable. The non-linear form of the convective term of the Navier–Stokes equations is considered to preserve the non-linear behaviour of the governing equations. The discretisation of the Navier–Stokes governing equations in the context of the pseudo-compressibility method of Chorin [23] and the solution of the governing equations in Eqs. (5)-(8) with respect to the primitive variables of interest in the succeeding pseudo-time level is performed in the following manner

(9) $$p_{i,j,k}^{n+1}=p_{i,j,k}^{n}+\beta \Delta \tau RHSP_{i,j,k}^{n},$$

(10) $$u_{i,j,k}^{n+1}=u_{i,j,k}^{n}+\Delta \tau RHSU_{i,j,k}^{n},$$

(11) $$v_{i,j,k}^{n+1}=v_{i,j,k}^{n}+\Delta \tau RHSV_{i,j,k}^{n},$$

(12) $$w_{i,j,k}^{n+1}=w_{i,j,k}^{n}+\Delta \tau RHSW_{i,j,k}^{n},$$

where *i*, *j* and *k* are the spatial (grid) indices, $RHSU_{i,j,k}^{n}$, $RHSV_{i,j,k}^{n}$, $RHSW_{i,j,k}^{n}$ the right-hand side of the momentum equations in the *x*-, *y*- and *z*-directions, respectively, *n* the previous pseudo-time level and n+1 the succeeding level. The variable $RHSP_{i,j,k}^{n}$ substitutes the right-hand side of the perturbed continuity equation Eq. (5) of the pseudo-compressibility method. Based on the numerical schemes presented for the spatial discretisation of the governing equations, the right-hand side variables of the Navier–Stokes system of equations Eqs. (5)-(8) are expanded as follows

(13) $$RHSP_{i,j,k}^{n}=-\left(\frac{u_{i,j,k}^{n}-u_{i-1,j,k}^{n}}{\Delta x}+\frac{v_{i,j,k}^{n}-v_{i,j-1,k}^{n}}{\Delta y}+\frac{w_{i,j,k}^{n}-w_{i,j,k-1}^{n}}{\Delta z}\right),$$

(14) $$RHSU_{i,j,k}^{n}=-\left(u_{i,j,k}^{n}\frac{u_{i,j,k}^{n}-u_{i-1,j,k}^{n}}{\Delta x}+v_{i,j,k}^{n}\frac{u_{i,j,k}^{n}-u_{i,j-1,k}^{n}}{\Delta y}+w_{i,j,k}^{n}\frac{u_{i,j,k}^{n}-u_{i,j,k-1}^{n}}{\Delta z}\right)-\frac{1}{\varrho }\frac{p_{i+1,j,k}^{n}-p_{i,j,k}^{n}}{\Delta x}+\nu \left(\frac{u_{i-1,j,k}^{n}-2u_{i,j,k}^{n}+u_{i+1,j,k}^{n}}{\Delta x^{2}}+\frac{u_{i,j-1,k}^{n}-2u_{i,j,k}^{n}+u_{i,j+1,k}^{n}}{\Delta y^{2}}+\frac{u_{i,j,k-1}^{n}-2u_{i,j,k}^{n}+u_{i,j,k+1}^{n}}{\Delta z^{2}}\right),$$

(15) $$RHSV_{i,j,k}^{n}=-\left(u_{i,j,k}^{n}\frac{v_{i,j,k}^{n}-v_{i-1,j,k}^{n}}{\Delta x}+v_{i,j,k}^{n}\frac{v_{i,j,k}^{n}-v_{i,j-1,k}^{n}}{\Delta y}+w_{i,j,k}^{n}\frac{v_{i,j,k}^{n}-v_{i,j,k-1}^{n}}{\Delta z}\right)-\frac{1}{\varrho }\frac{p_{i,j+1,k}^{n}-p_{i,j,k}^{n}}{\Delta y}+\nu \left(\frac{v_{i-1,j,k}^{n}-2v_{i,j,k}^{n}+v_{i+1,j,k}^{n}}{\Delta x^{2}}+\frac{v_{i,j-1,k}^{n}-2v_{i,j,k}^{n}+v_{i,j+1,k}^{n}}{\Delta y^{2}}+\frac{v_{i,j,k-1}^{n}-2v_{i,j,k}^{n}+v_{i,j,k+1}^{n}}{\Delta z^{2}}\right),$$

(16) $$RHSW_{i,j,k}^{n}=-\left(u_{i,j,k}^{n}\frac{w_{i,j,k}^{n}-w_{i-1,j,k}^{n}}{\Delta x}+v_{i,j,k}^{n}\frac{w_{i,j,k}^{n}-w_{i,j-1,k}^{n}}{\Delta y}+w_{i,j,k}^{n}\frac{w_{i,j,k}^{n}-w_{i,j,k-1}^{n}}{\Delta z}\right)-\frac{1}{\varrho }\frac{p_{i,j,k+1}^{n}-p_{i,j,k}^{n}}{\Delta z}+\nu \left(\frac{w_{i-1,j,k}^{n}-2w_{i,j,k}^{n}+w_{i+1,j,k}^{n}}{\Delta x^{2}}+\frac{w_{i,j-1,k}^{n}-2w_{i,j,k}^{n}+w_{i,j+1,k}^{n}}{\Delta y^{2}}+\frac{w_{i,j,k-1}^{n}-2w_{i,j,k}^{n}+w_{i,j,k+1}^{n}}{\Delta z^{2}}\right).$$

In the present investigation, a collocated grid arrangement is suggested for the elimination of the complications entailed in staggered grid arrangements. The pseudo-time stepping marching strategy is implemented through the utilisation of an explicit, third-order accurate Runge–Kutta scheme with total-variation diminishing (TVD) properties [70]:

(17) $$u^{⁎}=u^{n}+\Delta \tau RHS\left(u^{n}\right),$$

(18) $$u^{⁎⁎}=\frac{3}{4}u^{n}+\frac{1}{4}u^{⁎}+\Delta \tau RHS\left(u^{⁎}\right),$$

(19) $$u^{n+1}=\frac{1}{3}u^{n}+\frac{2}{3}u^{⁎⁎}+\frac{2}{3}\Delta \tau RHS\left(u^{⁎⁎}\right),$$

where Δ*τ* is the pseudo-time step and the *RHS* parameter indicates the right-hand side of the corresponding momentum equation. The utilisation of an explicit formulation for the time integration is justified on the basis of exploiting known information on the primitive variables propagation across the computational domain, for the determination of the variable values at the next pseudo-time step. In addition, the small pseudo-time step values generated in the proposed numerical approach are an important aspect of the explicit time integration formulations to capture high frequency solutions, despite the nonlinear characteristics of the problem originating from the convective term of the Navier–Stokes momentum equations [71]. For the estimation of the local pseudo-time step in each iteration, the following process is performed, initially the computational domain is scanned and the element with the highest local velocity magnitude is determined. Subsequently, the pseudo-time steps accounting for the inviscid and viscous effects of the Navier–Stokes equations are estimated, based on the velocity components at the domain element with the maximum local velocity magnitude [37], identified in the previous step of the implemented iterative process, as

(20) $$\Delta \tau_{inv}=\frac{CFL_{inv}\cdot \min\left(dx,dy,dz\right)}{\sqrt{u^{2}+v^{2}+w^{2}}+\sqrt{u^{2}+v^{2}+w^{2}+\beta }},$$

(21) $$\Delta \tau_{vis}=\frac{CFL_{vis}}{4\nu \cdot \min\left({\left(dx\right)}^{2},{\left(dy\right)}^{2},{\left(dz\right)}^{2}\right)}.$$

The parameters $CFL_{inv}$ and $CFL_{vis}$ are the inviscid and viscous Courant–Friedrichs–Lewy (CFL) numbers, the selection of which affects the overall performance of the numerical solution with respect to both the accuracy and convergence rates [72]. For two-dimensional flows, the parameters related to the *z*-direction are eliminated. After introducing a safety factor, γ∈[0,1] [73], the local pseudo-time step in each iteration is determined as the minimum between the estimated pseudo-time steps related to the inviscid and viscous parts of the momentum equations as

(22) $$\Delta \tau =\gamma \cdot \min\left(\Delta \tau_{inv},\Delta \tau_{vis}\right).$$

Note that under-relaxation methods were excluded during the implementation of the proposed numerical approach, for the constraint of the deviation of the primitive variables between two consecutive iterations. Ultimately, for the mathematical modelling of microfluidic flows characterised by extremely small length scales, the validity of the continuum hypothesis needs to be examined. The hypothesis allows for the examination of the individual atoms of the fluid in terms of a continuum model and can be validated through the comparison of the characteristic length of the examined system with the interatomic spacing of the fluid substances. The calculation of the average interatomic spacing of a fluid substance originates from the definition of the molecular volume and can be described [74] as

(23) $$\lambda_{fluid}={\left(\frac{M_{fluid}}{\varrho N_{A}}\right)}^{\frac{1}{3}},$$

where $M_{fluid}$ is the molar mass of the fluid substance and $N_{A}=6.02214\cdot 10^{23}$ $\left(mol^{-1}\right)$ the Avogadro constant. In this study, the continuum hypothesis is validated for each microfluidic computational example presented.

### 2.4 Mathematical formulation of the pressure outlet boundary condition

The mathematical formulation for the treatment of the pressure outlet boundary condition to overcome the limitation of the pseudo-compressibility method in viscous-dominant, incompressible flows, constitutes the basis for the proposed numerical approach at very-low Reynolds numbers (*Re* << 1). The approach relies on the qualitative and quantitative characteristics of the information exploited for the pressure propagation across the channel length. For the determination of the pressure-field, characteristics-based (CB) schemes only consider the information on the pressure from the adjacent cell faces of the boundaries [29] and require the extrapolation of the pressure values. In the context of the employed standard finite difference method (FDM) for the spatial discretisation of the primitive variables, the imposition of high-order derivative Neumann-type conditions at the domain boundaries further allows for the examination of nodes nonadjacent to the boundaries. The employment of backward approximations for the high-order derivatives of the Neumann-type condition at the outlet section, provides important information on the pressure propagation upwind of the domain boundary, without requiring the application of any extrapolation methods. Through the proposed treatment of the pressure outlet boundary condition, a greater perspective on the information propagation inside the microfluidic channel is gained, allowing for the proper estimation of the pressure-field and the development of the flow-field across the length of the channel. The first step for the implementation of the proposed numerical approach is the solution of the perturbed continuity equation in Eq. (3) for the determination of an initial pressure-field in the pseudo-compressibility formulation, with respect to the pressure gradient normal to the wall as

(24) $$\frac{\partial p}{\partial n}=-\beta \Delta \tau \left[\frac{\partial }{\partial n}\nabla \cdot u\right].$$

The enforcement of divergence-free characteristics on the velocity-field at the computational domain boundaries [41], eliminates the right-hand side of Eq. (24). Thus, the first-order derivative of the pressure variable normal to the wall boundary is equal to zero, i.e., a first-order derivative Neumann-type condition. Considering the first-order derivative to be equal to zero, higher-order derivatives are equal to zero as well. Hence, higher-order Neumann-type conditions can be employed for the determination of the primitive variable at the boundaries of the computational domain. For the derivation of the high-order approximations for the first-, second- and third-order derivative Neumann-type boundary conditions, a mathematical formulation suggested by Fornberg [75] for arbitrarily spaced grids is applied as follows

(25) $$\frac{\partial^{m}p}{\partial x_{i}^{m}}=\sum_{j=jmin}^{jmax}\frac{N_{j}p\left(x_{i}+j\Delta x_{i}\right)}{D\Delta x_{i}^{m}}+O\left(\Delta x_{i}^{a}\right)=0,$$

where *m* is the order of the derivative, *p* the hydrodynamic pressure, $x_{i}$ and $\Delta x_{i}$ the cartesian coordinate and grid spacing vectors, respectively, $j_{min}$ and $j_{max}$ are the extreme indices related to the order of the approximation, $N_{j}$ the numerator and *D* the denominator for the rational coefficients and *a* is the order of accuracy of the approximation. In addition to the discretisation error arising from the employment of the finite difference method (FDM) for the discretisation of the fluid flow governing equations and the boundary conditions of the primitive variables, the truncation error of the approximations for the Neumann-type boundary conditions has to be considered. The term $O\left(\Delta x_{i}^{a}\right)$ appearing on the right-hand side of Eq. (25) indicates the truncation error of the employed approximation. These types of error are accounted for in the present analysis as possible sources of error and are included in the quantified percentage deviations of the numerical solutions obtained against analytical solutions (see Section 3). The value of the ratio $N_{j}/D$ determines the weight coefficient for the approximation of a primitive variable in each node of the computational domain (see Table 1). For instance, the third-order backward approximation $O(\Delta x^{3})$ for the second-order derivative Neumann-type condition in a rectangular domain at the outlet can be formulated as

(26) $${\frac{\partial^{2}p}{\partial x^{2}}|}_{imax,j,k}^{n}=\frac{104p_{imax-1,j,k}^{n}-114p_{imax-2,j,k}^{n}+56p_{imax-3,j,k}^{n}-11p_{imax-4,j,k}^{n}}{35\Delta x_{imax}^{2}}+O\left(\Delta x_{imax}^{3}\right)=0,$$

where imax is the grid index in *x*-direction at the outlet section, and *n* superscript denotes the previous pseudo-time level in an explicit finite difference solution of the pressure-velocity coupling set of Eqs. (9)–(12). By multiplying Eq. (26) with $\Delta x_{imax}^{2}$, pressure is directly computed along the outlet boundary at a given pseudo-time level as

(27) $$p_{imax,j,k}^{n}=\frac{104p_{imax-1,j,k}^{n}-114p_{imax-2,j,k}^{n}+56p_{imax-3,j,k}^{n}-11p_{imax-4,j,k}^{n}}{35}.$$

In addition to the number of interior points of the computational domain considered for the determination of the pressure-field boundary condition, it is important to highlight the influence of the weight coefficients for the estimation of the primitive variable value at the node of interest. For instance, the third-order backward approximation $O(\Delta x^{3})$ for the second-order derivative Neumann-type condition in Eq. (26) allows for the estimation of the pressure at the outlet boundary, accounting for information on the pressure propagation across the length of the domain from four internal computational nodes. Similarly, the fourth-order backward approximation $O(\Delta x^{4})$ for the first-order derivative Neumann-type condition also considers four internal nodes for the estimation of the pressure at the outlet boundary as

(28) $${\frac{\partial p}{\partial x}|}_{imax,j,k}^{n}=\frac{48p_{imax-1,j,k}^{n}-36p_{imax-2,j,k}^{n}+16p_{imax-3,j,k}^{n}-3p_{imax-4,j,k}^{n}}{25\Delta x_{imax}}+O\left(\Delta x_{imax}^{4}\right)=0,$$

therefore, pressure can be accurately predicted at the computational nodes of the outlet section of the channel as

(29) $$p_{imax,j,k}^{n}=\frac{48p_{imax-1,j,k}^{n}-36p_{imax-2,j,k}^{n}+16p_{imax-3,j,k}^{n}-3p_{imax-4,j,k}^{n}}{25}.$$

However, the differentiation of the two formulations arises from the generation of the finite difference weights and the order of accuracy for each approximation. The solution algorithm described in [75] for the determination of the weight coefficients at the computational nodes of interest is based on Lagrange interpolation polynomial and justifies the accuracy of the derivative approximations. The estimation of the weight coefficients, and as a result the approximated value at the domain boundary, depends on the grid spacing and the order of the derivative. Different derivative order yields different values of the weight coefficients, yet same order of accuracy for the approximation. In this way, the information on the pressure propagation across the computational domain varies in the case where Neumann-type conditions of different derivative order are employed for the estimation of pressure on the domain boundaries. The weight coefficients for the up to fourth-order backward approximations for the first-, second- and third-order derivative of the Neumann-type conditions are summarised in Table 1. The treatment of the pressure boundary condition in the proposed numerical approach, is only applied at the outlet section, controlling the overall development of the flow-field across the length of the channel. The values of the pressure variable at the outlet boundary are estimated based on the information propagation for the corresponding primitive variable along the computational domain, allowing for the proper estimation of the pressure-field. For the rest of the computational domain boundaries, the fundamental first-order approximations O(Δx) for the first-order derivative Neumann-type conditions are employed. Note that the discretisation schemes employed in this work present specific limitations with respect to the type of meshes that it can be applied on. In the present form, the numerical formulation can only be applied in structured meshes. Therefore, the geometric configurations investigated in the present work concern two- and three-dimensional, straight, microfluidic channel flows of rectangular cross-section in Cartesian coordinates. The work of Fornberg [75] constitutes the basis of the numerical approach for the generation of finite difference formulations regards arbitrarily spaced grids.

**Table 1:** Backward finite difference approximations for the first-, second- and third-order derivative [75].

*n*	*a*	*j*_*min*_	*j*_*max*_	*D*	*N*_−6_	*N*_−5_	*N*_−4_	*N*_−3_	*N*_−2_	*N*_−1_	*N*_0_
1	1	-1	0	1						-1	1
1	2	-2	0	2					1	-4	3
1	3	-3	0	6				-2	9	-18	11
1	4	-4	0	12			3	-16	36	-48	25
2	1	-2	0	1					1	-2	1
2	2	-3	0	1				-1	4	-5	2
2	3	-4	0	12			11	-56	114	-104	35
2	4	-5	0	12		-10	61	-156	214	-154	45
3	1	-3	0	1				-1	3	-3	1
3	2	-4	0	2			3	-14	24	-18	5
3	3	-5	0	4		-7	41	-98	118	-71	17
3	4	-6	0	8	15	-104	307	-496	461	-232	49

As shown in Section 3, the imposition of either Dirichlet-type or high-order derivatives for the Neumann-type pressure boundary condition at the outlet section of the examined channel flow geometries, is the key factor to overcome the limitations of Chorin's [23] pseudo-compressibility method in very-low Reynolds number, incompressible flows (*Re* << 1). The results bring the treatment of the boundary conditions for the pressure variable into focus, rather than the numerical formulation of the pseudo-compressibility method, as an origin for its computational inefficiency.

### 2.5 Numerical solution procedure

For the solution of the stationary, incompressible Navier–Stokes governing equations Eqs. (3)-(4) in the framework of Chorin's [23] pseudo-compressibility method and the employment of the appropriate formulations for the treatment of the outlet boundary condition for the pressure, the following solution algorithm is implemented in iterative manner:

1. Prior to the initialisation of the iteration process, the velocity- and pressure-field variables are set equal to the conditions of the Initial-Value Problem (IVP). The first step of the iteration process is the imposition of the boundary conditions for the primitive variables, with the exception of the outlet boundary conditions for the velocity-field.
2. The pseudo-time step value is estimated, based on the velocity components at the domain element with the maximum local velocity magnitude, the pseudo-compressibility parameter and the Courant–Friedrichs–Lewy (CFL) numbers for the inviscid and viscous parts of the Navier–Stokes equations, as described in Section 2.3.
3. An internal iteration process over the stages of the explicit third-order Runge–Kutta method for the pseudo-temporal discretisation is embedded in the external iteration process. The calculation of the primitive variables for the determination of the velocity- and pressure-field is performed within this internal iteration process. For the explicit solution of the perturbed continuity equation in Eq. (3) of the pseudo-compressibility method, a loop over the internal computational domain is implemented. The values of the divergence of the velocity-field and the updated values of the pressure-field are obtained. Latter to the solution of the perturbed continuity equation in Eq. (3) for the corresponding stage of the Runge–Kutta method, the proposed numerical treatment for the outlet boundary condition of the updated pressure-field is performed. As discussed in Section 2.4, the imposition of either Dirichlet-type or higher-order derivative Neumann-type pressure-outlet boundary conditions is proposed.
4. A loop over the internal computational domain is also required for the solution of the momentum governing equations in Eq. (4). The convective and diffusion terms of the governing equations are being computed and the updated values of the velocity-field are obtained. For the determination of the pressure gradient term, the pressure-field values recovered from the numerical solution of the perturbed continuity equation of the pseudo-compressibility method are employed. The boundary conditions for the velocity-field at the outlet section are now updated.
5. The solution of the perturbed continuity equation in Eq. (3) and the momentum equations in Eq. (4) for each node of the internal computational domain is performed for each stage of the third-order Runge–Kutta method, for the temporal discretisation and time-marching in pseudo-time. The internal iteration process is terminated.
6. The values of the resulted numerical solution are then stored, and the iteration algorithm is repeated until the estimated error between two consecutive values of the variable of interest becomes smaller than a specified threshold value. The external iteration process is terminated allowing for the post-processing of the obtained results.

**Figure 1:**
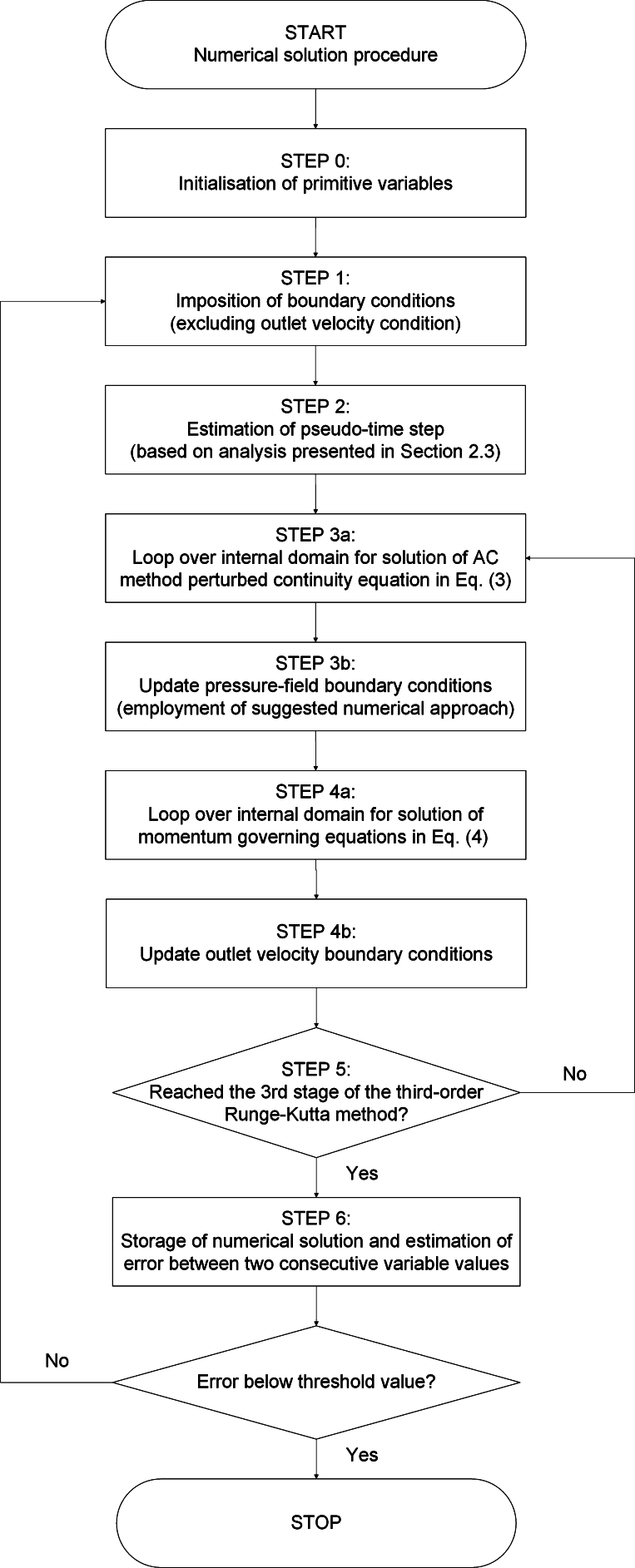
Algorithm flowchart of numerical approach for high-order treatment of pressure boundary conditions.

The algorithm flowchart describing the proposed numerical approach presented above is depicted in Fig. 1. As shown in Section 3, the application of the aforementioned algorithm for the employment of either Dirichlet-type or high-order derivative Neumann-type boundary conditions at the outlet section of the investigated microfluidic channels is essential to overcome the computational limitations of the Artificial Compressibility method of Chorin [23] in very-low Reynolds number flows (*Re* << 1). In the next section, a discussion will be made on the effect of the employment of high-order derivative Neumann-type pressure outflow boundary conditions on the flow-field development. Through the comparison of the numerically computed axial velocity component solutions with analytical solutions available in the literature, the necessity of a high-order treatment for the pressure outlet boundary condition to allow for the flow-field development will be exhibited. The obtained numerical results will also indicate the imposition of low-order derivative Neumann-type boundary conditions for the pressure variable at the microfluidic channel outlet section as inappropriate, producing percentage errors against the corresponding analytical solutions up to approximately 85% and 95% in the cases of the two- and three-dimensional, straight, microfluidic channel flows, respectively. The attention that this numerical investigation brings to the treatment of the pressure boundary conditions rather than the numerical formulation of the pseudo-compressibility method is fundamental to overcome its computational limitations in very-low Reynolds number, incompressible flows. An integral part to comprehend the effect of low-order derivative Neumann-type boundary conditions on the flow-field deterioration is the qualitative and quantitative characteristics of the information propagation of the pressure variable across the channel length, controlled by the pressure outlet boundary condition, as discussed in Section 2.4. The numerical results exhibiting the importance of an appropriate treatment for the pressure outlet boundary condition will be shown in the next section.

## 3 Results and discussion

In this section, the results of the proposed numerical approach for the mathematical modelling and the numerical solution of very-low Reynolds number (*Re* << 1), incompressible, hydrodynamic channel flows are presented. Initially, the formulation for the analytical solutions against which the numerical results are validated, is reported. Then, the details on the numerical investigation for the determination of the pseudo-compressibility parameter are described, in addition to the performed grid refinement study. Lastly, the results of the numerical simulations are presented and discussed. For the validation of the proposed numerical approach in very-low Reynolds numbers ranging from $10^{-1}$ to $10^{-4}$, the computational examples characterising steady, incompressible, flows in straight, microfluidic channels are as follows: a) two-dimensional flows governed by the Navier–Stokes, b) two-dimensional flows governed by the Oseen equations, and c) three-dimensional flows governed by the Navier–Stokes in cartesian coordinates. For the performance of the required numerical simulations, an in-house solver for serial computing in the C language has been implemented on an Intel(R) Core(TM) i7-10700 at 2.90 GHz computer with 32GB RAM memory.

Following the introduction to the governing equations of the Artificial Compressibility (AC) method of Chorin [23] and the details for the mathematical formulations of the pressure outlet boundary conditions, the results of the proposed numerical approach for the high-order treatment of the pressure outlet boundary condition to overcome the computational limitations of the AC method in very-low Reynolds number flows, will be presented. By the end of this section, relevant conclusions will be obtained for the validity of the currently proposed approach to extend the computational capabilities of the pseudo-compressibility method of Chorin [23] in strongly viscous flows. Initially, it is shown that the imposition of Dirichlet-type and high-order derivative Neumann-type boundary conditions for the pressure variable at the outlet section of the microfluidic channels performs satisfactorily for the accurate description of the flow-field development in the investigated pressure-driven flows. In contrast, the employment of first-order derivative Neumann-type pressure outlet boundary conditions appears to deteriorate the fluid flow-field development and produces non-physical results. A useful conclusion is derived from the numerical results presented in this section, regards the imposition of low-order approximations for high-order Neumann-type conditions. It is shown that regardless of the finite difference approximation order, focus should be adverted to the order of the employed derivatives for the Neumann-type boundary conditions. For instance, the utilisation of fourth-order approximations for the first-order derivative Neumann-type pressure outlet boundary condition results in the deterioration of the flow-field development, whereas the imposition of first-order approximations for the second-order derivative Neumann-type condition allows for the accurate development of the investigated flow-field. Comprehensive details on the numerical implementation details and the simulation results for the examined computational examples are reported subsequently in this section.

### 3.1 Two-dimensional, straight, microfluidic channel flow

#### 3.1.1 Analytical solution of the plane Hagen–Poiseuille flow

**Figure 2:**
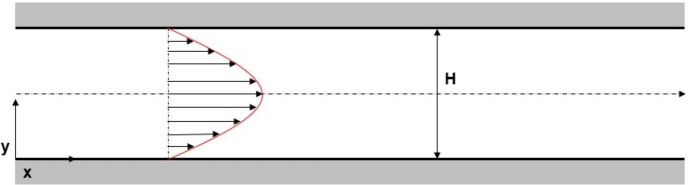
Two-dimensional, straight, microfluidic channel geometry.

The two-dimensional, straight, microfluidic channel flow governed by the Navier–Stokes is validated against the analytical solution of the plane Hagen–Poiseuille flow, else a laminar, incompressible, viscous flow between two parallel, stationary flat plates. The dimensions of the microfluidic channel are given as follows, $L=10^{-4}$ (*m*) is the length and $H=10^{-5}$ (*m*) the height of the two-dimensional configuration. The selection of the channel and rectangular cross-section dimensions is based on the classification of channel flows with respect to the magnitude of the hydraulic diameter, $D_{h}$. In the case of a two-dimensional flow between two parallel, stationary flat plates, the hydraulic diameter is defined as $D_{h}=2H$. The resulted hydraulic diameter of the two-dimensional, microfluidic channel is therefore estimated as $D_{h}=20$ (μm). In the work of Mehendale et al. [76], a hydraulic diameter equal to 20 (μm) characterises the channel as “microchannel”. A channel described by the same geometric dimensions is also classified as a “microchannel” in the work of Kandlikar and Grande [77]. The selection of the two-dimensional, straight, channel dimensions relies on the certainty that the investigated flows are “microfluidic” (Fig. 2). The working fluid is water at 4^∘^, with ρ=1000 $\left(kg/m^{3}\right)$ and μ=0.00102 (Pa⋅s). For the pressure-driven Poiseuille flow, the axial velocity component is only considered to be a function of the radial direction, u=u(y). The velocity components in the remaining cartesian coordinates directions are eliminated, v,w=0. No-slip boundary conditions for the axial velocity are imposed on the top and bottom wall boundaries of the two-dimensional computational domain.

The Boundary Value Problem (BVP) for the two-dimensional, pressure-driven, Poiseuille flow reads as

(30) $$0=-\frac{1}{\mu }\frac{dp}{dx}+\frac{\partial^{2}u}{\partial y^{2}},$$

(31) u(x,0)=0,u(x,H)=0,

where *H* is the height of the microfluidic channel and pressure is only a function of the axial direction. The axial velocity distribution of the hydrodynamically fully-developed, pressure-driven Poiseuille flow is obtained [78] as

(32) $$u\left(y\right)=\frac{1}{2\mu }\frac{\Delta p}{L}\left(y^{2}-yH\right),$$

where *μ* is the dynamic viscosity of the fluid, Δ*p* is the pressure difference between the inlet and the outlet section of the channel and *L* is the length of the microfluidic channel. The volumetric flow rate between the parallel, stationary flat plates is calculated by integrating the velocity distribution in Eq. (32) across the microfluidic channel height as

(33) $$Q=W\int_{0}^{H}u\left(y\right)dy=W\frac{\Delta pH^{3}}{12\mu L},$$

where *W* is the width of the microfluidic channel. The pressure difference between the inlet and the outlet section of the two-dimensional, straight, microfluidic channel is estimated based on the relationship, U=Q/A, correlating the uniform mean flow velocity (*U*), the volumetric flow rate in Eq. (33) and the cross-section of the channel (*A*), as

(34) $$\Delta p=\frac{12\mu U}{H^{2}}.$$

Through the determination of the velocity distribution from Eq. (32), the axial velocity profile at the outlet section of the microfluidic channel can be analytically estimated and compared against the numerical results of the pseudo-compressibility method in very-low Reynolds numbers. As discussed, the dimensionless Reynolds number accounts for the ratio of the inertia forces to the viscous forces of the fluidic system and is determined as [79] $Re=(D_{h}U)/\nu$, where $D_{h}$ is the hydraulic diameter. The accuracy of the numerical solution is evaluated based on three distinct error metrics, estimated at the outlet section of the two-dimensional, straight, microfluidic channel geometry. The mean percentage error is defined as the mean percentage deviation of the numerically predicted axial velocity profile from the corresponding analytically computed velocity profile. The maximum percentage error is defined as the maximum percentage deviation across the obtained axial velocity profiles, and the centreline percentage error as the percentage deviation of the centreline point of the axial velocity profile estimated numerically, from the analytical solution.

#### 3.1.2 Numerical implementation details

The details for the numerical implementation of the two-dimensional, straight, microfluidic channel flow governed by the Navier–Stokes and the Oseen equations are discussed. Boundary conditions for the primitive variables of pressure and velocity are imposed on all four boundaries of the computational domain. At the inlet section of the microfluidic channel, the axial velocity component is set equal to the uniform mean flow velocity, the velocity component in the *y*-direction is set equal to zero, and a first-order forward approximation for the first-derivative Neumann-type condition is imposed for the pressure-field. At the wall boundaries, no-slip boundary conditions are imposed for the velocity components, while a first-order forward and a first-order backward approximation for the first-derivative Neumann-type condition is imposed on the bottom and top wall boundary, respectively. Lastly, first-order backward approximations for the first-derivative Neumann-type conditions are imposed for the velocity components at the outlet section of the two-dimensional, microfluidic channel, whilst Dirichlet-type conditions and up to fourth-order backward approximations are constructed for the first-, second- and third-order derivative Neumann-type conditions for the pressure variable. The Dirichlet-type conditions examined are specified values of the pressure-field equal to either zero, or the dynamic pressure, $p=(\rho U^{2})/2$. The initialisation of the primitive variables across the computational domain is performed by setting the axial velocity component equal to the uniform mean flow velocity, the velocity component in the *y*-direction equal to zero and the pressure-field variable equal to the analytically calculated pressure drop across the channel length, as shown in Eq. (34). The numerical solution of the two-dimensional, straight, microfluidic channel flow governed by the Navier–Stokes and the Oseen equations is validated against the analytical solution obtained by the plane Hagen–Poiseuille flow as reported in Eq. (32). For each of the two computational examples, 14 different mathematical formulations for the pressure boundary condition at the outlet section of the microfluidic channel at 4 different Reynolds numbers are investigated, yielding a total of 56 numerical simulations.

#### 3.1.3 Two-dimensional, straight, microfluidic channel flow governed by the Navier–Stokes equations

**Figure 3:**
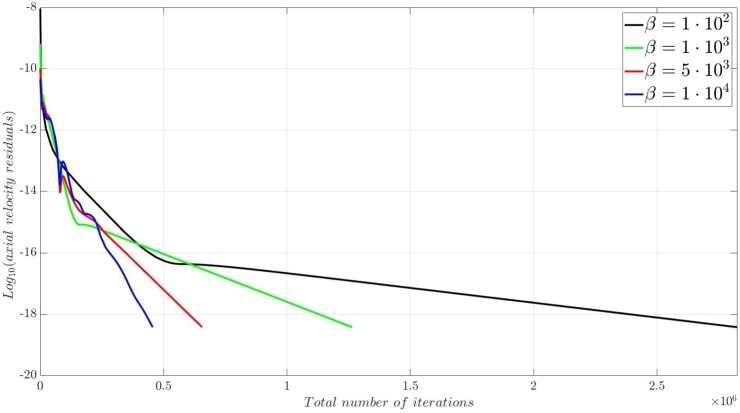
Axial velocity residuals for different pseudo-compressibility parameter *β* values at *Re* = 10^−1^ with a grid size of 15*x*10.

**Figure 4:**
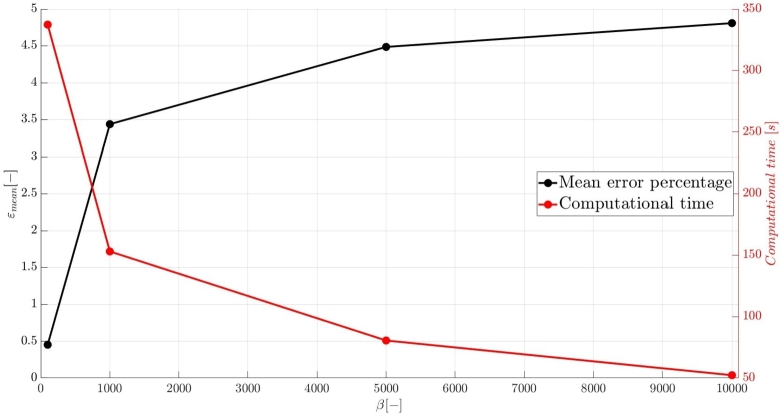
Mean error percentage and computational time for different pseudo-compressibility parameter *β* values at *Re* = 10^−1^ with a grid size of 15*x*10.

A numerical investigation for the determination of the pseudo-compressibility parameter and the grid refinement study for the appropriate selection of the grid size are now presented. The significance of the pseudo-compressibility parameter on the overall performance of the numerical solver was discussed. In the proposed approach, an appropriate value for the AC parameter is determined through the numerical investigation of different parameter values, at a fixed Reynolds number. Following the investigation of the behaviour of the residuals for the axial velocity component, the criteria for the parameter selection involve the mean error percentage between the numerical and analytical axial velocity profile in the outlet section, and the computational time required until convergence to pseudo steady-state. Four different values of the AC parameter are investigated ($\beta =10^{2},10^{3},5\cdot 10^{3},10^{4}$), in the case of $Re=10^{-1}$, a coarse grid of 15x10 nodes and a Dirichlet-type pressure boundary condition equal to zero at the outlet section of the examined, two-dimensional geometry. Following the numerical experiments for the determination of an appropriate pseudo-compressibility parameter *β* value, a systematic mesh sensitivity analysis for the two-dimensional, straight, microfluidic channel flow governed by the Navier–Stokes equations is performed, based on the determined value. As it can be seen in Fig. 3, the convergence history for different values of the artificial compressibility (AC) parameter, indicates a disproportional relationship between the values assigned to the pseudo-compressibility parameter and the number of iterations required to converge to pseudo steady-state. For the AC parameter values of 10^2^, 10^3^, $5\cdot 10^{3}$ and 10^4^, the required numbers of iterations are 2825878, 1264424, 656186 and 455538, respectively. Note that the axial velocity component residuals for a pseudo-compressibility parameter equal to 10^2^ and 10^3^ exhibit a monotonic behaviour, whilst the convergence history for parameter values of $5\cdot 10^{3}$ and 10^4^ are oscillatory. In Fig. 4, an increase in the mean error percentage between the numerical and analytical axial velocity profile at the outlet section of the channel, and a decrease in the computational time required to achieve pseudo steady-state is observed, as the AC parameter increases. The numerical simulation with a parameter value of 10^2^ requires 337.283 (*s*) to reach pseudo steady-state, whilst 153.069 (*s*) are required for an AC parameter equal to 10^3^, 80.553 (*s*) for a parameter equal to $5\cdot 10^{3}$ and 52.3 (*s*) for a parameter value of 10^4^. The mean error percentages are 0.4519%, 3.4380%, 4.4859% and 4.8088%, respectively. The results indicate the value of 10^3^ for the pseudo-compressibility parameter, as an appropriately selected value to balance the accuracy and computational efficiency of the numerical solution. In the proposed approach, and considering the disproportional correlation which has been reported in numerical experiments in the literature between the AC parameter and the Reynolds number, the reduction of an order of magnitude in the Reynolds number value, yields an increase of an order of magnitude for the pseudo-compressibility parameter *β*. A summary of the simulation details with respect to numerous convergence indicators obtained from the numerical experiments performed for the determination of an appropriate pseudo-compressibility parameter *β* is reported in Table 8. The value of the pseudo-compressibility parameter, *β*, and the designated tolerance *ϵ* required for convergence of the numerical solution to pseudo steady-state at each of the four different, investigated Reynolds number values, are shown in Table 2. Both the inviscid and viscous Courant–Friedrichs–Lewy (CFL) numbers are set equal to 0.015, whereas the safety factor *γ* for the determination of the pseudo-time step in each iteration is set equal to 0.9.

**Table 2:** Simulation parameters of two-dimensional, straight, microfluidic channel flow governed by the Navier–Stokes equations at different Reynolds numbers.

	Simulation parameters			
*Re*	10^−1^	10^−2^	10^−3^	10^−4^
*β*	10^3^	10^4^	10^5^	10^6^
*ε*	10^−8^	10^−9^	10^−10^	10^−11^

The grid refinement study and the estimation of the discretisation error adopted throughout this numerical investigation is based on the computation of the Grid Convergence Index (GCI) [80], [81]. Three different sets of grids of 126, 551 and 5191 total number of cells are examined for the computations, whilst the metric of interest is the mean value of the axial velocity component at the outlet section of the two-dimensional, straight, microfluidic channel. In the definition of the GCI, the safety factor is set equal to 1.25. The results showed that the grid convergence is achieved for all cases, in each of the Reynolds numbers ranging from $10^{-1}$ to $10^{-4}$, based on the simulation parameters at different grid levels. In Table 3, the sample calculations of the discretisation error for $Re=10^{-1}$ and a Dirichlet-type pressure boundary condition equal to zero at the outlet section of the examined, two-dimensional geometry are reported. The grid refinement factor, defined as the intermediate to the fine mesh ratio, is 3.0694, when a value greater than 1.3 is proposed in the literature [81]. The apparent order of the grid convergence method $p_{GCI}$ is estimated to be 2.4318 and the numerical uncertainty in the fine grid solution, i.e., the Grid Convergence Index (GCI) is 0.1438%.

**Table 3:** Computation of discretisation error.

	*ϕ*= mean value of axial velocity
	profile at outlet section
*N*_1_,*N*_2_,*N*_3_	5191, 551, 126
*r*_21_	3.0694
*r*_32_	2.0912
*ϕ*_1_	4.8892 ⋅ 10^−3^
*ϕ*_2_	4.8088 ⋅ 10^−3^
*ϕ*_3_	4.3776 ⋅ 10^−3^
*p*_*GCI*_	2.4318
$\phi_{ext}^{21}$	4.8948 ⋅ 10^−3^
$e_{a}^{21}$	1.6444%
$e_{ext}^{21}$	0.1149%
$GCI_{fine}^{21}$	0.1438%

Note that despite of the fact that the GCI indicates the utilisation of the intermediate mesh, the fine mesh has been selected for the performed simulations, due to the enhanced accuracy of the numerical solutions, varying from 0.5−1.0%. The fine mesh consists of 180x30 nodes, comprising the computational domain and the domain boundaries.

**Figure 5:**
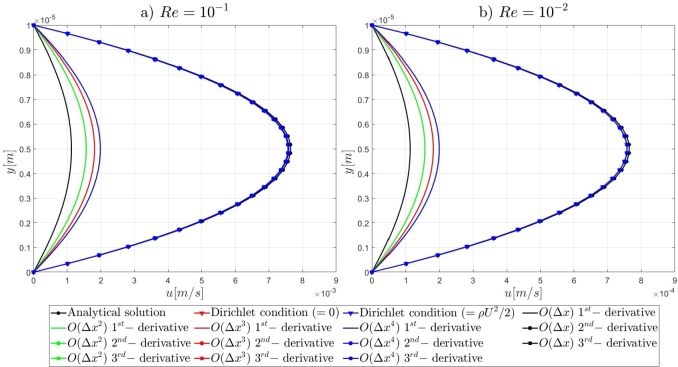
Numerical solution of axial velocity profiles against analytical solution of Eq. (32) for different pressure outlet boundary conditions at a) *Re* = 10^−1^ and b) *Re* = 10^−2^ with a grid size of 180*x*30.

**Figure 6:**
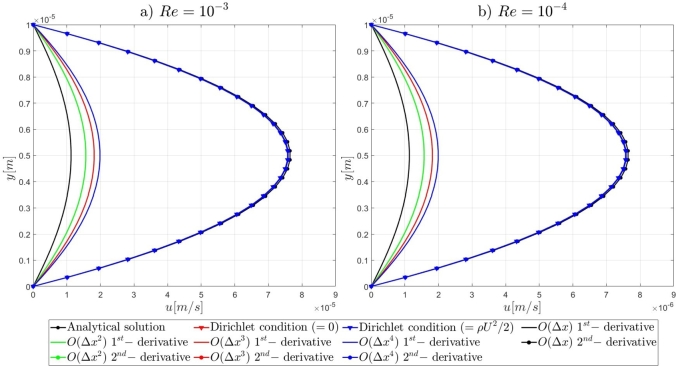
Numerical solution of axial velocity profiles against analytical solution of Eq. (32) for different pressure outlet boundary conditions at a) *Re* = 10^−3^ and b) *Re* = 10^−4^ with a grid size of 180*x*30.

The first computational example for the validation of the proposed approach for the mathematical modelling and the numerical solution of steady, incompressible channel flows in very-low Reynolds numbers ranging from $10^{-1}$ to $10^{-4}$, is a two-dimensional, straight, microfluidic channel flow governed by the Navier–Stokes equations. As discussed, two Dirichlet-type conditions and up to fourth-order backward approximations are constructed for the first-, second- and third-order derivative Neumann-type conditions for the pressure at the outlet section of the channel. As it can be seen in Figure 5, Figure 6, the numerical simulations for each of the four different Reynolds number indicate the imposition of the two Dirichlet-type and the up to fourth-order approximations for the second- and third-order derivative Neumann-type conditions as appropriate, to overcome the computational limitations of the pseudo-compressibility method in flows characterised by Reynolds number below O(1). For $Re=10^{-1}$, three distinct error metrics range from 0.710% to 1.003%; for $Re=10^{-2}$, the error ranges from 0.822% to 0.891%; while for $Re=10^{-3}$ and $Re=10^{-4}$, the range is from 0.727% to 0.942% and from 0.748% to 0.983%, respectively. The maximum, mean and centreline percentage errors between the numerically computed axial velocity profile and the analytical solution at the outlet section of the channel reported in Eq. (32) are below 1%, which is acceptable. Note, however, that the numerical simulations for $Re\leq 10^{-3}$, employing third-order derivative Neumann-type pressure boundary conditions at the outlet section, failed to converge to a numerical solution, despite the employed approximation order.

Furthermore, Figure 5, Figure 6 highlight that, regardless of the order of the approximation, numerical simulations employing first-order derivative Neumann-type conditions do not allow the flow-field development, yielding unphysical results and confirming the stiffness of the pseudo-compressibility method in such viscous-dominant flows. Characteristically, for each of the examined Reynolds numbers and backward approximations of the first-order derivative Neumann-type conditions, all three percentage errors range from 73.939% to 85.606%. It is thereby shown that, the treatment proposed in the present study for the pressure outlet boundary condition of a two-dimensional, straight, microfluidic channel flow governed by the Navier–Stokes equations, is appropriate to overcome the limitations the pseudo-compressibility method entangles in very-low Reynolds number flows (*Re* << 1). Further insight on the numerical solutions indicates that higher-order approximations of the second- and third-order derivative Neumann-type conditions do not, most definitely, provide more accurate results than the lower-order approximations, despite the differentiations observed in the computational time and number of iterations required to converge to pseudo steady-state. Therefore, the treatment of the outlet boundary condition for the pressure variable facilitated to overcome the limitations of the pseudo-compressibility method of Chorin [23], does not necessarily require high-order truncation error approximations, merely high-order derivatives for the Neumann-type pressure outflow boundary condition.

In addition to the accuracy of the produced results, the overall computational efficiency of a numerical solution is characterised by the computational time and the number of iterations performed to converge to pseudo steady-state. The computational time required for the numerical simulations imposing pressure outlet boundary conditions that allow the flow-field development across the length of the computational domain, ranges from 385.44 (min) to 428.30 (min) for $Re=10^{-1}$, from 123.31 (min) to 130.32 (min) for $Re=10^{-2}$, from 156.89 (min) to 165.92 (min) for $Re=10^{-3}$ and from 327.27 (min) to 370.09 (min) for $Re=10^{-4}$. The total number of iterations required to achieve pseudo steady-state ranges from 7257281 to 7861701, 2312807 to 2425750, 2968790 to 3043043 and 6156762 to 6942872 iterations, respectively. On the contrary, the numerical simulations employing approximations for the first-order derivative Neumann-type condition required significantly less computational time and number of iterations to converge to pseudo steady-state and achieve the designated tolerance for the axial velocity residuals, as indicated for each examined Reynolds number. However, as shown, low-order derivative Neumann-type conditions do not allow the flow-field development and are inappropriate to overcome the computational stiffness of the pseudo-compressibility method, regardless of the approximation order. The simulation metrics, in addition to the three error percentages for the evaluation of the numerical solutions in each Reynolds number, are summarised in Table 4, Table 5, Table 6, Table 7.

**Table 4:** Simulation results for two-dimensional, straight, microfluidic channel flow governed by the Navier–Stokes equations against analytical solution of Eq. (32) for different pressure outlet boundary conditions at *Re* = 10^−1^ with a grid size of 180*x*30.

Reynolds number (*Re* = 10^−1^)		Simulation metrics		Percentage error against Eq. (32)		
Type	Condition	Comp. time (min)	Iterations	Maximum	Mean	Centreline
Dirchlet	0.0	383.50	7 257 929	0.710%	0.710%	0.710%
	*ρU*^2^/2	385.44	7 257 281	0.710%	0.710%	0.710%
Neumann	*O*(Δ*x*)	147.92	2 795 169	85.278%	85.606%	85.278%
(1^*st*^-derivative)	*O*(Δ*x*^2^)	164.97	3 109 283	79.543%	79.891%	79.543%
	*O*(Δ*x*^3^)	174.38	3 291 823	76.294%	76.656%	76.295%
	*O*(Δ*x*^4^)	184.23	3 419 607	74.046%	74.416%	74.047%
Neumann	*O*(Δ*x*)	416.35	7 854 254	1.003%	1.003%	1.003%
(2^*nd*^-derivative)	*O*(Δ*x*^2^)	416.08	7 857 392	1.003%	1.003%	1.003%
	*O*(Δ*x*^3^)	415.73	7 857 727	1.003%	1.003%	1.003%
	*O*(Δ*x*^4^)	415.07	7 857 836	1.003%	1.003%	1.003%
Neumann	*O*(Δ*x*)	428.30	7 861 701	1.003%	1.003%	1.003%
(3^*rd*^-derivative)	*O*(Δ*x*^2^)	424.76	7 860 476	1.003%	1.003%	1.003%
	*O*(Δ*x*^3^)	416.29	7 860 023	1.003%	1.003%	1.003%
	*O*(Δ*x*^4^)	421.49	7 859 700	1.003%	1.003%	1.003%

**Table 5:** Simulation results for two-dimensional, straight, microfluidic channel flow governed by the Navier–Stokes equations against analytical solution of Eq. (32) for different pressure outlet boundary conditions at *Re* = 10^−2^ with a grid size of 180*x*30.

Reynolds number (*Re* = 10^−2^)		Simulation metrics		Percentage error against Eq. (32)		
Type	Condition	Comp. time (min)	Iterations	Maximum	Mean	Centreline
Dirchlet	0.0	123.31	2 312 821	0.822%	0.822%	0.822%
	*ρU*^2^/2	126.39	2 312 807	0.822%	0.822%	0.822%
Neumann	*O*(Δ*x*)	49.14	924 672	85.260%	85.589%	85.261%
(1^*st*^-derivative)	*O*(Δ*x*^2^)	55.52	1 046 402	79.515%	79.865%	79.516%
	*O*(Δ*x*^3^)	58.43	1 101 933	76.261%	76.623%	76.262%
	*O*(Δ*x*^4^)	41.53	772 220	73.951%	74.320%	73.952%
Neumann	*O*(Δ*x*)	128.37	2 422 764	0.891%	0.890%	0.891%
(2^*nd*^-derivative)	*O*(Δ*x*^2^)	128.35	2 424 001	0.891%	0.890%	0.891%
	*O*(Δ*x*^3^)	128.60	2 424 171	0.891%	0.890%	0.891%
	*O*(Δ*x*^4^)	128.06	2 424 228	0.891%	0.890%	0.891%
Neumann	*O*(Δ*x*)	129.46	2 425 750	0.891%	0.891%	0.891%
(3^*rd*^-derivative)	*O*(Δ*x*^2^)	130.32	2 425 353	0.891%	0.890%	0.891%
	*O*(Δ*x*^3^)	128.74	2 425 189	0.891%	0.890%	0.891%
	*O*(Δ*x*^4^)	129.11	2 425 060	0.891%	0.890%	0.891%

**Table 6:** Simulation results for two-dimensional, straight, microfluidic channel flow governed by the Navier–Stokes equations against analytical solution of Eq. (32) for different pressure outlet boundary conditions at *Re* = 10^−3^ with a grid size of 180*x*30.

Reynolds number (*Re* = 10^−3^)		Simulation metrics		Percentage error against Eq. (32)		
Type	Condition	Comp. time (min)	Iterations	Maximum	Mean	Centreline
Dirchlet	0.0	165.92	3 043 043	0.942%	0.947%	0.942%
	*ρU*^2^/2	161.72	3 043 043	0.942%	0.947%	0.942%
Neumann	*O*(Δ*x*)	103.96	1 962 100	85.264%	85.591%	85.264%
(1^*st*^-derivative)	*O*(Δ*x*^2^)	104.05	1 967 871	79.525%	79.873%	79.526%
	*O*(Δ*x*^3^)	94.84	1 739 329	76.266%	76.629%	76.267%
	*O*(Δ*x*^4^)	92.77	1 729 955	74.013%	74.384%	74.014%
Neumann	*O*(Δ*x*)	156.89	2 968 790	0.730%	0.728%	0.730%
(2^*nd*^-derivative)	*O*(Δ*x*^2^)	157.91	2 969 851	0.729%	0.727%	0.729%
	*O*(Δ*x*^3^)	157.62	2 969 927	0.729%	0.727%	0.729%
	*O*(Δ*x*^4^)	157.68	2 969 952	0.729%	0.727%	0.729%

**Table 7:** Simulation results for two-dimensional, straight, microfluidic channel flow governed by the Navier–Stokes equations against analytical solution of Eq. (32) for different pressure outlet boundary conditions at *Re* = 10^−4^ with a grid size of 180*x*30.

Reynolds number (*Re* = 10^−4^)		Simulation metrics		Percentage error against Eq. (32)		
Type	Condition	Comp. time (min)	Iterations	Maximum	Mean	Centreline
Dirchlet	0.0	327.65	6 156 762	0.970%	0.983%	0.970%
	*ρU*^2^/2	327.27	6 156 762	0.970%	0.983%	0.970%
Neumann	*O*(Δ*x*)	232.34	4 378 593	85.190%	85.521%	85.190%
(1^*st*^-derivative)	*O*(Δ*x*^2^)	206.64	3 891 628	79.427%	79.780%	79.428%
	*O*(Δ*x*^3^)	205.92	3 882 879	76.201%	76.566%	76.202%
	*O*(Δ*x*^4^)	196.07	3 680 941	73.939%	74.315%	73.941%
Neumann	*O*(Δ*x*)	366.16	6 939 088	0.763%	0.751%	0.763%
(2^*nd*^-derivative)	*O*(Δ*x*^2^)	370.09	6 942 699	0.760%	0.748%	0.760%
	*O*(Δ*x*^3^)	368.11	6 942 821	0.760%	0.748%	0.760%
	*O*(Δ*x*^4^)	369.89	6 942 872	0.760%	0.748%	0.760%

#### 3.1.4 Two-dimensional, straight, microfluidic channel flow governed by the Oseen equations

The second computational example presented in the current work is the two-dimensional, straight, microfluidic channel flow governed by the Oseen equations. Viscous-dominant flows are also referred in the literature as creeping flows, or Stokes flows [82]. Stokes equations are a modification of the general, incompressible Navier-Stokes governing equations, assuming that the dimensionless Reynolds number approaches the zero-limit value and that the effect of the external fluidic body forces, sinks and sources is ultimately neglected [83]. Numerous studies have attempted to investigate the behaviour of wall-bounded Stokes flows, either from a theoretical, or a practical perspective [18], [84], [85], in addition to the mathematical modelling of creeping flows for various geometrical configurations [86], [87], [88]. Low- and very-low Reynolds number channel flows governed by the Oseen equations have also been previously investigated in the literature [89], [90]. The two-dimensional, steady-state, incompressible Oseen formulation consists of the mass conservation (continuity) and the momentum governing equations and can be expanded in scalar form as

(35) $$\frac{\partial u}{\partial x}+\frac{\partial v}{\partial y}=0,$$

(36) $$U\frac{\partial u}{\partial x}=-\frac{1}{\varrho }\frac{\partial p}{\partial x}+\nu \left(\frac{\partial^{2}u}{\partial x^{2}}+\frac{\partial^{2}u}{\partial y^{2}}\right),$$

(37) $$U\frac{\partial v}{\partial x}=-\frac{1}{\varrho }\frac{\partial p}{\partial y}+\nu \left(\frac{\partial^{2}v}{\partial x^{2}}+\frac{\partial^{2}v}{\partial y^{2}}\right),$$

where again *U* is the uniform mean flow velocity. As it can be seen, the differentiation of Oseen's formulation with respect to the Stokes equations is the partial inclusion of the linearised convective acceleration in the momentum equations Eqs. (36) and (37). For the Oseen's equations the linearised convective acceleration is expressed in terms of an inner product of the uniform mean flow velocity and the gradient of the local fluid velocity [91]. The governing equations of the two-dimensional Oseen flows in the context of the pseudo-compressibility method of Chorin [23] have been formulated as described below and its mathematical application first time presented in this numerical study. The pseudo-compressibility formulation algorithm for the steady-state, incompressible Oseen equations consists of the perturbed continuity equation and the pseudo-transient Oseen momentum equations which reads in scalar form as

(38) $$\frac{1}{\beta }\frac{\partial p}{\partial \tau }+\frac{\partial u}{\partial x}+\frac{\partial v}{\partial y}=0,$$

(39) $$\frac{\partial u}{\partial \tau }+U\frac{\partial u}{\partial x}=-\frac{1}{\varrho }\frac{\partial p}{\partial x}+\nu \left(\frac{\partial^{2}u}{\partial x^{2}}+\frac{\partial^{2}u}{\partial y^{2}}\right),$$

(40) $$\frac{\partial v}{\partial \tau }+U\frac{\partial v}{\partial x}=-\frac{1}{\varrho }\frac{\partial p}{\partial y}+\nu \left(\frac{\partial^{2}v}{\partial x^{2}}+\frac{\partial^{2}v}{\partial y^{2}}\right),$$

where the examined Reynolds number range and the employed boundary conditions are the same as in the case of the two-dimensional, straight, microfluidic channel benchmark problem described in Subsection 3.1.2. An analysis for the determination of the pseudo-compressibility parameter and the grid refinement study are presented. Similar to the example of the two-dimensional, straight, microfluidic channel flow governed by the Navier–Stokes equations, four different values of the pseudo-compressibility parameter are investigated ($\beta =10^{2},10^{3},5\cdot 10^{3},10^{4}$), in the case of $Re=10^{-1}$, a coarse grid of 15x10 nodes and a Dirichlet-type pressure boundary condition equal to zero at the outlet section of the examined, two-dimensional geometry. Following the numerical experiments for the determination of an appropriate pseudo-compressibility parameter *β* value, a systematic mesh sensitivity analysis for the two-dimensional, straight, microfluidic channel flow governed by the Oseen equations is performed, based on the determined value.

**Figure 7:**
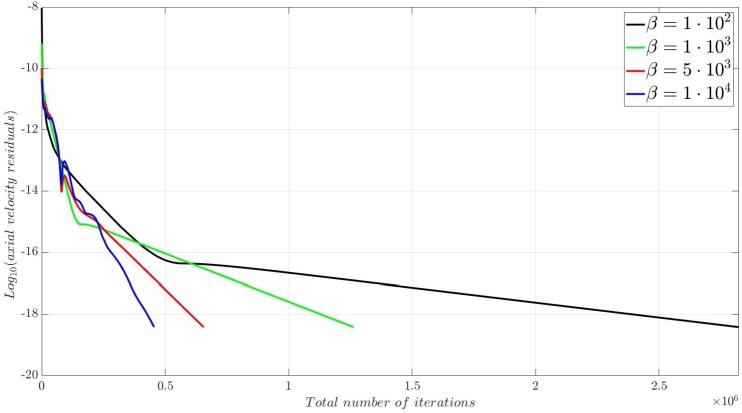
Axial velocity residuals for different pseudo-compressibility parameter *β* values at *Re* = 10^−1^ with a grid size of 15*x*10.

**Figure 8:**
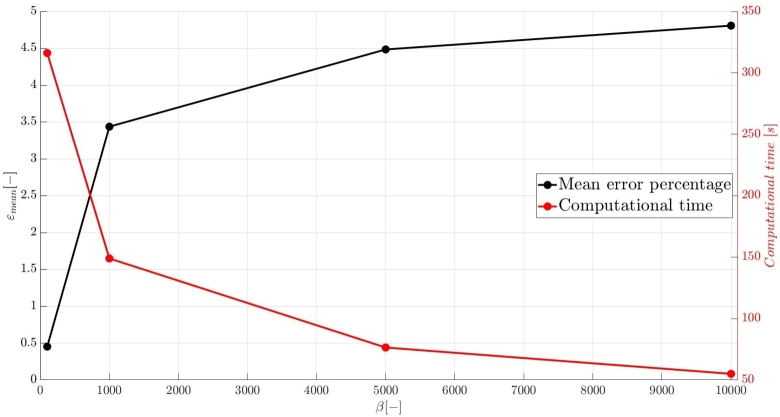
Mean error percentage and computational time for different pseudo-compressibility parameter *β* values at *Re* = 10^−1^ with a grid size of 15*x*10.

In Fig. 7, the convergence history for the different values of the pseudo-compressibility parameter is shown. A disproportional relationship between the value of the pseudo-compressibility parameter and the number of iterations required to achieve convergence to pseudo steady-state, is observed. In particular, a pseudo-compressibility parameter value of 10^2^ requires 2822603 iterations for convergence, while 1263366, 655731 and 455195 iterations are required for the convergence of the numerical solution utilising a pseudo-compressibility parameter value of 10^3^, $5\cdot 10^{3}$ and 10^4^, respectively. The residuals for the axial velocity component for a pseudo-compressibility parameter equal to 10^2^ and 10^3^ are characterised by a monotonic behaviour, in contrast to the corresponding residuals for parameter values of $5\cdot 10^{3}$ and 10^4^ which exhibit an oscillatory behaviour. In Fig. 8, the metrics upon the determination of the value of the AC parameter is based, are presented. Similar to the previous computational example, an increase of the pseudo-compressibility parameter value is followed by an increase in the mean error percentage between the numerically and analytically computed axial velocity profiles at the outlet section of the channel, in addition to a decrease in the computational time of the obtained numerical solution. The computational time required for a numerical simulation with a prescribed AC parameter value of 10^2^ to reach pseudo steady-state is 316.157 (*s*), whereas 148.908 (*s*) are required for a parameter value of 10^3^, 76.386 (*s*) for a parameter value of $5\cdot 10^{3}$ and 54.981 (*s*) for the pseudo-compressibility parameter equal to 10^4^. The mean error percentages are 0.4524%, 3.4367%, 4.4845% and 4.8075%, respectively. As a result and, in terms of the overall efficiency of the numerical solution, the value of 10^3^ is selected for the AC parameter. Accounting for the disproportional relationship of the pseudo-compressibility parameter and the Reynolds number, an order of magnitude decrease on the latter, results in an order of magnitude increase on the former. For the numerical solution of the two-dimensional, Oseen flow in a straight, microfluidic channel, identical simulation parameters with the previous flow governed by the Navier–Stokes equations are set, as shown in Table 2.

As discussed, the differentiation of the Navier–Stokes equations with the Oseen formulation is the linearised convective acceleration appearing in the momentum equations. In very-low Reynolds number flows, such as the ones investigated in the present study, the impact of the convective term on the fluid characteristics is limited. Therefore, trivial differences are expected between the two-dimensional, straight, microfluidic channel flow computational examples governed by the Navier–Stokes and the Oseen equations. These minor differences of the two computational examples are exhibited in Figure 3, Figure 7 for the axial velocity residuals and the total number of iterations required until numerical convergence to a pseudo steady-state. In Figure 4, Figure 8 with respect to the computational time until convergence and the mean error percentage of the numerically computed axial velocity profile against the analytical solution reported in Eq. (32) at the outlet section of the microfluidic channel, in the case of the fluid flow governed by the Navier–Stokes and the Oseen equations, respectively. The deviations of the simulation metrics between the two-dimensional fluid flow computational examples investigated in the present study are summarised in Table 8.

**Table 8:** Simulation metrics of computational examples of two-dimensional, straight, microfluidic channel flows governed by the Navier–Stokes and the Oseen equation residuals for different pseudo-compressibility parameter values at *Re* = 10^−1^ with a grid size of 15*x*10.

	Number of iterations		Comp. time (s)		Mean error percentage against Eq. (32)	
Governing equations	Navier–Stokes	Oseen	Navier–Stokes	Oseen	Navier–Stokes	Oseen
*β* = 10^2^	2 825 878	2 822 603	337.283	316.157	0.4519%	0.4524%
*β* = 10^3^	1 264 424	1 263 366	153.069	148.908	3.4380%	3.4367%
*β* = 5 ⋅ 10^3^	656 186	655 731	80.553	76.386	4.4859%	4.4845%
*β* = 10^4^	455 538	455 195	52.300	54.981	4.8088%	4.8075%

The grid refinement study and the estimation of the discretisation error for the two-dimensional, straight, microfluidic channel flow governed by the Oseen equations, is performed in a similar manner to the previous benchmark problem. Three different grid levels of 126, 551 and 5191 total number of cells are investigated, whereas the mean value of the axial velocity component at the outlet section of the two-dimensional geometry is utilised as the critical metric for the performance of the mesh convergence and sensitivity analysis. The safety factor is set equal to 1.25. As expected, grid convergence is observed for all cases across the examined Reynolds number range. Similarly, in Table 9 the calculations of the discretisation error for $Re=10^{-1}$ and a Dirichlet-type pressure boundary condition equal to zero at the outlet section of the channel are reported. The grid refinement factor is equal to 3.0694 and the apparent order of the grid convergence method $p_{GCI}$ is 2.4337. The Grid Convergence Index (GCI) is estimated to be 0.1436%.

**Table 9:** Computation of discretisation error.

	*ϕ*= mean value of axial velocity
	profile at outlet section
*N*_1_,*N*_2_,*N*_3_	5191, 551, 126
*r*_21_	3.0694
*r*_32_	2.0912
*ϕ*_1_	4.8892 ⋅ 10^−3^
*ϕ*_2_	4.8089 ⋅ 10^−3^
*ϕ*_3_	4.3776 ⋅ 10^−3^
*p*_*GCI*_	2.4337
$\phi_{ext}^{21}$	4.8948 ⋅ 10^−3^
$e_{a}^{21}$	1.6424%
$e_{ext}^{21}$	0.1146%
$GCI_{fine}^{21}$	0.1436%

The grid refinement study indicates the utilisation of the intermediate mesh. Nonetheless, the fine mesh has been selected, due to the fact that the errors between the numerical and the analytical solution of the axial velocity profiles at the outlet section of the channel exhibit improved accuracy, ranging from 0.5% to 1.0%. Accordingly, the investigated computational mesh consists of 180x30 nodes which includes the internal domain nodes and the domain boundaries.

**Figure 9:**
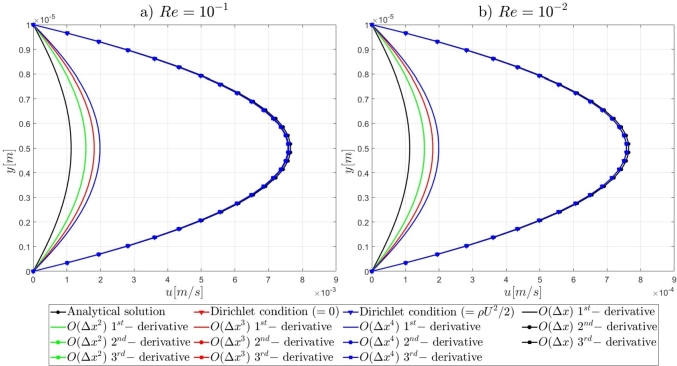
Numerical solution of axial velocity profiles against analytical solution of Eq. (32) for different pressure outlet boundary conditions at a) *Re* = 10^−1^ and b) *Re* = 10^−2^ with a grid size of 180*x*30.

The numerical results of the second benchmark problem are now presented. In Fig. 9, the numerical solution of the axial velocity profiles at the outlet section of the two-dimensional channel is validated against the analytical solution of the plane Poiseuille flow in Eq. (32), for different types of mathematical formulations for the pressure-field outlet boundary condition. As in the previous computational example, the imposition of Dirichlet-type and up to fourth-order approximations for the second- and third-order derivative Neumann-type conditions at the outlet section of the channel, allows for the appropriate flow-field development across the length of the computational domain, for each of the examined Reynolds numbers. Characteristically, the percentage errors for $Re=10^{-1}$ range from 0.709% to 1.002%, whereas for $Re=10^{-2}$ the errors range from 0.822% to 0.891%. Note that for $Re\leq 10^{-3}$, the numerical results obtained for the stationary, incompressible flow governed by the Oseen equations, are identical to those of the flow governed by the Navier–Stokes equations, hence, not reported. As discussed, the difference of the Oseen formulation in comparison with the Navier–Stokes equations, is the linearised convective acceleration in the momentum equations. Inevitably, in such low Reynolds number flows, characterised by the relative predominance of the viscous over the inertia forces, the significance of the convective term is strictly limited. For each simulation performed, the maximum, mean and centerline percentage errors defining the deviation between the numerical and the analytical solution of the axial velocity profile at the outlet section of the microfluidic channel are close to 1%.

The numerical results obtained again from the imposition of low-order approximations for the high-order derivative Neumann-type conditions, are comparable to the corresponding results obtained from the imposition of the higher-order approximations for the second- and third-order derivatives. This leads to the conclusion that the high-order treatment of the pressure outlet boundary condition for the Oseen flow is not restricted to high-order approximations for the second- and third-order derivative Neumann-type conditions, but low-order approximations are also found to perform satisfactorily. Similar to the previous computational example, simulations employing third-order derivative Neumann-type conditions for the pressure-field at the outlet section of the channel, failed to converge to a numerical solution, for $Re\leq 10^{-3}$. In Fig. 9, it is also seen that, first-derivative Neumann-type conditions for the pressure at the outlet section of the channel, yield unphysical numerical solutions. Taking into consideration the fact that, regardless of the Reynolds number and the order of the backward approximation for the first-derivative Neumann-type condition, the three distinct percentage errors range from 73.939% to 85.591%, the limitations of the pseudo-compressibility method in very-low Reynolds numbers (*Re* << 1) are again verified for this type of conditions. The results indicate the proposed numerical approach for the mathematical formulation of the boundary conditions for the pressure-field at the outlet section of a two-dimensional, straight, microfluidic channel flow governed by the Oseen equations, as appropriate, to exceed the limitations of the employed pseudo-compressibility method of Chorin [23].

The convergence histories of the numerical solutions for the axial velocity profiles at the outlet section of the two-dimensional channel are analysed both in terms of computational time, and total number of iterations to converge to pseudo steady-state. Initially, the simulation metrics of the numerical solutions imposing Dirichlet-type and second- and third-order derivatives Neumann-type conditions, that allow the development of the flow-field across the length of the channel, are reported. For $Re=10^{-1}$, the computational time required to achieve pseudo steady-state ranges from 408.12 (min) to 459.61 (min), while for $Re=10^{-2}$ the range is from 128.58 (min) to 141.02 (min), from 169.35 (min) to 178.85 (min) for $Re=10^{-3}$ and from 348.16 (min) to 398.43 (min) for $Re=10^{-4}$. The corresponding ranges for the number of iterations required to reach pseudo steady-state vary from 7252453 to 7861208 iterations for $Re=10^{-1}$ and from 2312662 to 2425715 for $Re=10^{-2}$. As discussed, the simulation metrics for $Re\leq 10^{-3}$, with the exception of the computational times required for the simulations to be performed, are identical to the metrics of the previous computational example. The numerical solution, characterised by the computational time and the number of iterations required to converge to pseudo steady-state, in addition to the percentage errors produced in comparison with the analytical solution of the plane Poiseuille flow for $Re>10^{-3}$, are summarised in Table 10, Table 11.

**Table 10:** Simulation results for two-dimensional, straight, microfluidic channel flow governed by the Oseen equations against analytical solution of Eq. (32) for different pressure outlet boundary conditions at *Re* = 10^−1^ with a grid size of 180*x*30.

Reynolds number (*Re* = 10^−1^)		Simulation metrics		Percentage error against Eq. (32)		
Type	Condition	Comp. time (min)	Iterations	Maximum	Mean	Centreline
Dirchlet	0.0	408.12	7 253 101	0.709%	0.709%	0.709%
	*ρU*^2^/2	411.69	7 252 453	0.709%	0.709%	0.709%
Neumann	*O*(Δ*x*)	158.54	2 797 087	85.249%	85.578%	85.250%
(1^*st*^-derivative)	*O*(Δ*x*^2^)	174.94	3 110 991	79.520%	79.869%	79.521%
	*O*(Δ*x*^3^)	185.01	3 293 422	76.274%	76.636%	76.275%
	*O*(Δ*x*^4^)	193.92	3 421 139	74.028%	74.398%	74.029%
Neumann	*O*(Δ*x*)	435.88	7 853 735	1.002%	1.002%	1.002%
(2^*nd*^-derivative)	*O*(Δ*x*^2^)	426.35	7 856 885	1.002%	1.002%	1.002%
	*O*(Δ*x*^3^)	443.90	7 857 221	1.002%	1.002%	1.002%
	*O*(Δ*x*^4^)	459.61	7 857 330	1.002%	1.002%	1.002%
Neumann	*O*(Δ*x*)	424.17	7 861 208	1.002%	1.002%	1.002%
(3^*rd*^-derivative)	*O*(Δ*x*^2^)	418.16	7 859 979	1.002%	1.002%	1.002%
	*O*(Δ*x*^3^)	433.49	7 859 522	1.002%	1.002%	1.002%
	*O*(Δ*x*^4^)	455.82	7 859 196	1.002%	1.002%	1.002%

**Table 11:** Simulation results for two-dimensional, straight, microfluidic channel flow governed by the Oseen equations against analytical solution of Eq. (32) for different pressure outlet boundary conditions at *Re* = 10^−2^ with a grid size of 180*x*30.

Reynolds number (*Re* = 10^−2^)		Simulation metrics		Percentage error against Eq. (32)		
Type	Condition	Comp. time (min)	Iterations	Maximum	Mean	Centreline
Dirchlet	0.0	133.56	2 312 676	0.822%	0.822%	0.822%
	*ρU*^2^/2	128.58	2 312 662	0.822%	0.822%	0.822%
Neumann	*O*(Δ*x*)	50.48	924 734	85.258%	85.586%	85.258%
(1^*st*^-derivative)	*O*(Δ*x*^2^)	59.18	1 046 433	79.513%	79.862%	79.514%
	*O*(Δ*x*^3^)	61.76	1 101 941	76.259%	76.621%	76.260%
	*O*(Δ*x*^4^)	42.83	772 301	73.949%	74.319%	73.950%
Neumann	*O*(Δ*x*)	130.07	2 422 728	0.891%	0.890%	0.891%
(2^*nd*^-derivative)	*O*(Δ*x*^2^)	132.15	2 423 966	0.891%	0.890%	0.891%
	*O*(Δ*x*^3^)	141.02	2 424 135	0.891%	0.890%	0.891%
	*O*(Δ*x*^4^)	133.34	2 424 193	0.891%	0.890%	0.891%
Neumann	*O*(Δ*x*)	131.91	2 425 715	0.891%	0.890%	0.891%
(3^*rd*^-derivative)	*O*(Δ*x*^2^)	131.60	2 425 319	0.891%	0.890%	0.891%
	*O*(Δ*x*^3^)	132.11	2 425 154	0.891%	0.890%	0.891%
	*O*(Δ*x*^4^)	132.06	2 425 025	0.891%	0.890%	0.891%

### 3.2 Three-dimensional, straight, microfluidic channel flow

#### 3.2.1 Analytical solution for the outlet velocity distribution

**Figure 10:**
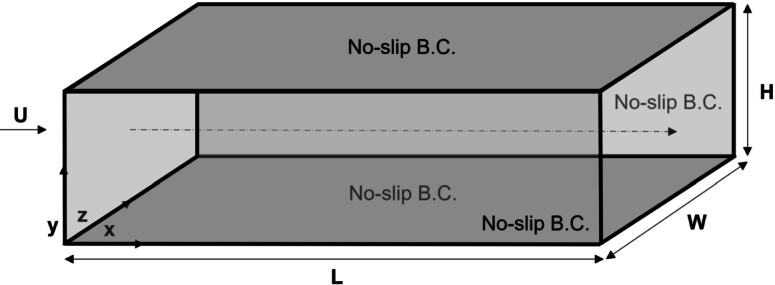
Three-dimensional, straight, microfluidic channel geometry.

The computational example of the three-dimensional, straight, microfluidic channel flow governed by the Navier–Stokes equations, is validated against the analytical solution of a stationary, incompressible, pressure-driven, hydrodynamically fully-developed, viscous flow through a rectangular cross-section channel. The dimensions of the rectangular cross-section are given as follows, $H=10^{-5}$ (*m*), $W=10^{-5}$ (*m*), and the hydraulic diameter is defined as, $D_{h}=2HW/(H+W)$. Again, the selection of the channel and rectangular cross-section dimensions relies on the channels classification with respect to the magnitude of the hydraulic diameter $D_{h}$. The resulted hydraulic diameter of the three-dimensional, microfluidic channel is computed as $D_{h}=10$ (μm). According to the classification of Mehendale et al. [76], the channel is therefore characterised as a “microchannel”, whilst Kandlikar and Grande [77] classify the investigated configuration as a “transitional microchannel”. In any case, the selection of the three-dimensional, straight, channel dimensions assures that the investigated flows are “microfluidic” (Fig. 10). Similar to the previously presented benchmark problems, the working fluid is water. The axial velocity component is a function of the *y*- and *z*-directions, u=u(y,z), whilst the velocity components in the directions of the remaining cartesian coordinates are neglected, v,w=0. For the derivation of an analytical solution for a fluid flow through a rectangular cross-section, no-slip boundary conditions are imposed for the axial velocity component on the left, right, bottom, and top wall boundaries. The Boundary Value Problem (BVP) for the pressure-driven flow through a rectangular cross-section reads as

(41) $$0=-\frac{1}{\mu }\frac{dp}{dx}+\frac{\partial^{2}u}{\partial y^{2}}+\frac{\partial^{2}u}{\partial z^{2}},$$

(42) u(0,z)=0,u(H,z)=0,

(43) u(y,0)=0,u(y,W)=0,

where *H* is the height and *W* the width of the rectangular cross-section and pressure is only a function of the axial direction. Following the definition of the computational domain as (y,z)=[0,H]x[0,W], an appropriate trial solution for the axial velocity component distribution in a Fourier sine-series form, satisfying the no-slip boundary conditions at the computational domain boundaries has been obtained in the literature by Marco and Han [92] and is given as

(44) $$u\left(y,z\right)=-\frac{16\frac{dp}{dx}}{\mu \pi^{4}}\sum_{\kappa ,\lambda \;\epsilon \;O^{+}}^{\infty }{\left[\kappa \;\lambda \left(\frac{\kappa^{2}}{H^{2}}+\frac{\lambda^{2}}{W^{2}}\right)\right]}^{-1}\sin\left(\frac{\kappa \pi y}{H}\right)\sin\left(\frac{\lambda \pi z}{W}\right).$$

The indices of summation *κ* and *λ*, belong to the set of positive, odd numbers κ,λ=1,3,5,… For the determination of the summation term appearing in Eq. (44), the first 1000 terms of the infinite sine-series are considered. Relying on the analytically computed axial velocity distribution in Eq. (44), the volumetric flow rate through the rectangular cross-section is estimated through the integration of u(y,z) across the rectangular cross-section dimensions as

(45) $$Q=\int_{0}^{H}\int_{0}^{W}u\left(y,z\right)dzdy=-\frac{64\frac{dp}{dx}}{\mu \pi^{6}}HW\sum_{\kappa ,\lambda \epsilon O^{+}}^{\infty }{\left[\kappa^{2}\lambda^{2}\left(\frac{\kappa^{2}}{H^{2}}+\frac{\lambda^{2}}{W^{2}}\right)\right]}^{-1},$$

whilst the axial pressure gradient is defined through the formulation U=Q/A, correlating the uniform mean flow velocity (*U*), the volumetric flow rate from Eq. (45) and the rectangular cross-section area defined as A=H⋅W, as

(46) $$\frac{dp}{dx}=-\frac{U\mu \pi^{6}}{64}\sum_{\kappa ,\lambda \;\epsilon \;O^{+}}^{\infty }\left[\kappa^{2}\lambda^{2}\left(\frac{\kappa^{2}}{H^{2}}+\frac{\lambda^{2}}{W^{2}}\right)\right].$$

On the assumption of a hydrodynamically fully-developed flow, the axial velocity distribution of the rectangular cross-section can be extended for the analytical solution of a three-dimensional, straight, microfluidic channel flow. The analysis allows for the comparison of the numerical results in the context of the pseudo-compressibility method in very-low Reynolds numbers, with the analytically calculated velocity distribution at the outlet section of the three-dimensional, microfluidic channel as reported in Eq. (44). In a similar manner to the two-dimensional computational examples, the quantification of the analysis is performed through the evaluation of five error metrics. The mean and maximum percentage errors are estimated for both the axial velocity distribution at the outlet section of the three-dimensional channel, and the axial velocity profile at the centreline of the middle cross-section of the outlet section. The errors are defined as the mean and maximum deviation of the numerical from the analytical obtained solutions, respectively. The centreline percentage error is defined as the deviation of the numerical solution at the midpoint of the outlet rectangular cross-section from the analytical solution of the velocity distribution reported in Eq. (44).

#### 3.2.2 Numerical implementation details

The numerical implementation details of the three-dimensional, straight, microfluidic channel flow governed by the Navier–Stokes equations are reported. Similar to the computational examples of the two-dimensional, straight, microfluidic channel flow governed by the Navier–Stokes and the Oseen equations, identical boundary conditions on all four boundaries of the computational domain are imposed for the primitive variables. In addition, the no-slip boundary condition for all three velocity components and a first-order forward and first-order backward approximation for the first-derivative Neumann-type condition is imposed for the pressure-field on the left and right wall boundary, respectively. The initial conditions for the primitive variables are similar to the conditions of the previous computational examples, coupled with the initialisation of the velocity component in the *z*-direction set equal to zero. For the mathematical modelling and the validation of the numerical solution of the examined flows, 4 different Reynolds numbers were studied for 14 different mathematical formulations for the boundary conditions of the pressure variable at the outlet section of the three-dimensional channel, accounting for a total of 56 numerical simulations.

#### 3.2.3 Three-dimensional, straight, microfluidic channel flow governed by the Navier–Stokes equations

The preliminary study for the determination of the pseudo-compressibility parameter and the grid refinement study of the third computational example are presented. In this benchmark problem, four different values of the pseudo-compressibility parameter are considered ($\beta =10^{2},10^{3},5\cdot 10^{3},10^{4}$), in the case of $Re=10^{-1}$. The simulations are performed on a grid of 10x10x10 nodes and a Dirichlet-type condition equal to zero, for the pressure at the outlet section of the three-dimensional, straight, microfluidic channel. Furthermore, latter to the investigation of the axial velocity component residuals, the mean percentage error between the numerically and analytically computed axial velocity component profiles from Eq. (44) and the computational time required to converge to pseudo steady-state, the obtained results are reported. Following the numerical experiments required for the estimation of a proper pseudo-compressibility parameter *β* value, a systematic mesh sensitivity analysis for the three-dimensional, straight, microfluidic channel flow governed by the Navier–Stokes equations is performed, based on the determined value.

**Figure 11:**
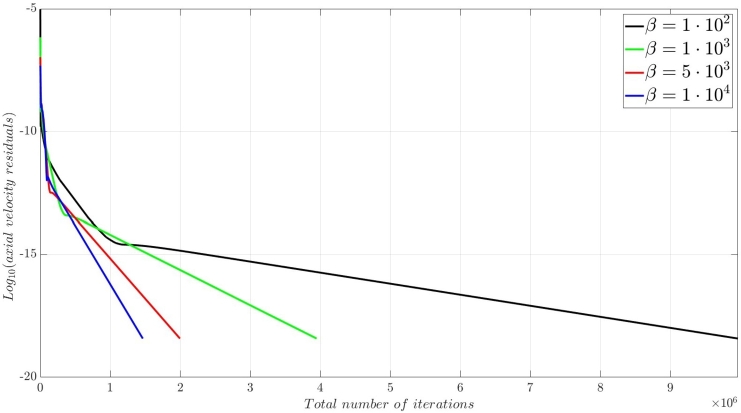
Axial velocity component residuals for different pseudo-compressibility parameter *β* values at *Re* = 10^−1^ with a grid size of 10*x*10*x*10.

**Figure 12:**
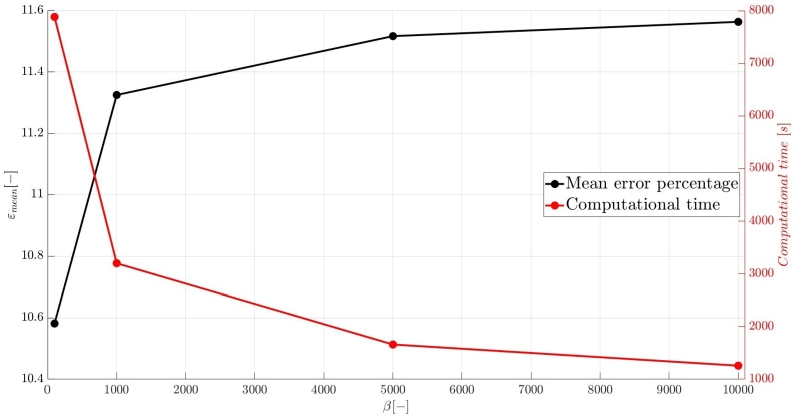
Mean error percentage and computational time for different AC parameter *β* values at *Re* = 10^−1^ with a grid size of 10*x*10*x*10.

As shown in Fig. 11, a disproportional relationship characterises the number of iterations required to reach pseudo steady-state and the value of the artificial compressibility (AC) parameter. For an AC parameter value equal to 10^2^, 9963713 iterations are required for convergence, whereas 3945914, 1994408 and 1463494 iterations are required for values of the pseudo-compressibility parameter equal to 10^3^, $5\cdot 10^{3}$ and 10^4^, respectively. Contrary to the computational examples of the two-dimensional, microfluidic channel flows governed by the Navier-Stokes and the Oseen equations, the axial velocity component residuals for all values of the pseudo-compressibility parameter exhibit a monotonic behaviour and no oscillations are observed. Furthermore, as seen in Fig. 12, a decrease in the computational time required to converge to pseudo steady-state and an increase of the mean error percentage, originate from an increase of the pseudo-compressibility value. The numerical simulation prescribing a value of 10^2^ for the AC parameter requires 7881.701 (*s*) to converge to pseudo steady-state. 3198.702 (*s*) are required for a parameter value equal to 10^3^, 1660.770 (*s*) for a parameter value equal to $5\cdot 10^{3}$ and 1258.495 (*s*) are required for a pseudo-compressibility value of 10^4^. The mean error percentages are equal to 10.5816%, 11.3253%, 11.5170% and 11.5637%, respectively. Considering the overall computational efficiency of the numerical solution, the designated value for the pseudo-compressibility parameter is set equal to $5\cdot 10^{3}$. Similar to the previous computational examples, the disproportionality between the AC parameter value and the Reynolds number, indicates an order of magnitude increment on the former, for an order of decrement on the latter, as lower Reynolds number flows are examined.

The simulation parameters for the three-dimensional, straight, microfluidic channel flow problem can now be determined. For different values of the Reynolds numbers characterising the fluid flow problem, the values of the pseudo-compressibility parameter *β* and the designated tolerance *ϵ* of the numerical solution to converge to pseudo steady-state are exhibited in Table 12. Similar to the two-dimensional computational examples, the inviscid and viscous Courant-Friedrichs-Lewy (CFL) numbers are set equal to 0.015 and the safety factor *γ* equal to 0.9.

**Table 12:** Simulation parameters of three-dimensional, straight, microfluidic channel flow governed by the Navier–Stokes equations at different Reynolds numbers.

	Simulation parameters			
*Re*	10^−1^	10^−2^	10^−3^	10^−4^
*β*	5 ⋅ 10^3^	5 ⋅ 10^4^	5 ⋅ 10^5^	5 ⋅ 10^6^
*ε*	10^−8^	10^−9^	10^−10^	10^−11^

As discussed, the grid refinement study for the estimation of the discretisation error is based on the Grid Convergence Index (GCI) method. The coarse, intermediate, and fine meshes investigated consist of 729, 5819 and 24389 cells, respectively. The critical variable for the determination of the GCI value is the mean value of the axial velocity profile at the centreline of the middle cross-section of the three-dimensional channel. The safety factor is equal to 1.25. Here, only the results for Reynolds number equal to $10^{-1}$ and a Dirichlet-type pressure boundary condition equal to zero at the outlet section of the examined geometry, are reported in Table 13. The GCI is calculated as 1.1381%, whereas the grid refinement factor is 1.6123 and the apparent order of the grid convergence method $p_{GCI}$ is 2.8336. Grid convergence is observed for all the simulations performed in the Reynolds number range from $10^{-1}$ to $10^{-4}$.

**Table 13:** Computation of discretisation error.

	*ϕ*= mean value of axial velocity profile
	at width middle of channel
*N*_1_,*N*_2_,*N*_3_	24 389, 5819, 729
*r*_21_	1.6123
*r*_32_	1.9985
*ϕ*_1_	9.3614 ⋅ 10^−3^
*ϕ*_2_	9.1167 ⋅ 10^−3^
*ϕ*_3_	7.0997 ⋅ 10^−3^
*p*_*GCI*_	2.8336
$\phi_{ext}^{21}$	9.4466 ⋅ 10^−3^
$e_{a}^{21}$	2.6139%
$e_{ext}^{21}$	0.9023%
$GCI_{fine}^{21}$	1.1381%

The grid refinement study indicates the utilisation of the fine mesh for the numerical solution of the three-dimensional, straight, microfluidic channel flow. Based on the grid refinement study, the fine mesh employed consists of 30x30x30 nodes across the internal nodes of computational domain in addition to the domain boundaries.

**Figure 13:**
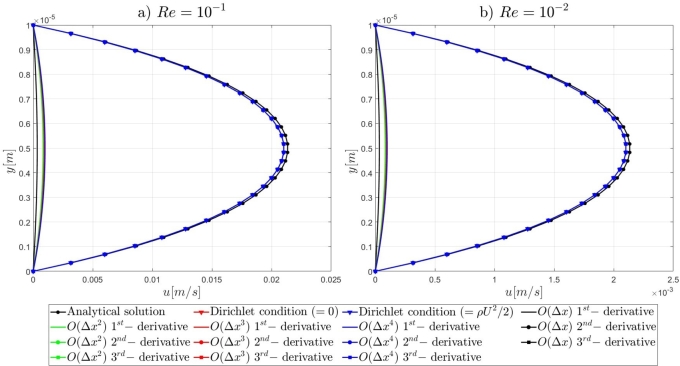
Numerical solution of axial velocity profiles against analytical solution of Eq. (44) for different pressure outlet boundary conditions at the centreline of the middle cross-section of the outlet section and a) *Re* = 10^−1^ and b) *Re* = 10^−2^ with a grid size of 30*x*30*x*30.

**Figure 14:**
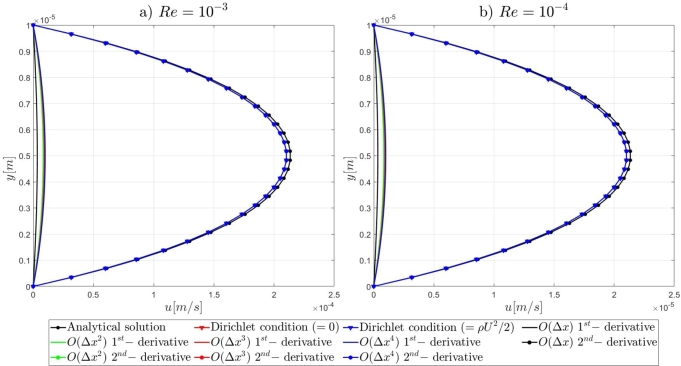
Numerical solution of axial velocity profiles against analytical solution of Eq. (44) for different pressure outlet boundary conditions at the centreline of the middle cross-section of the outlet section and a) *Re* = 10^−3^ and b) *Re* = 10^−4^ with a grid size of 30*x*30*x*30.

The numerical results for the three-dimensional, straight, microfluidic channel flow governed by the Navier–Stokes equations are now presented. For the evaluation of the accuracy of the produced results in different Reynolds numbers and pressure outlet boundary conditions, the axial velocity profiles at the centreline of the middle cross-section of the outlet section of the channel are validated in Figure 13, Figure 14, against the analytical solution of a fully-developed, pressure-driven flow through a rectangular cross-section reported in Eq. (44). As shown, in numerical simulations characterised by the imposition of Dirichlet-type and high-order derivative Neumann-type conditions for the pressure at the outlet section of the channel, the flow-field development is rather accurate. More precisely, all five error metrics investigated in this analysis range from 1.495% to 1.542% for $Re=10^{-1}$, from 1.506% to 1.525% for $Re=10^{-2}$ from 1.501% to 1.525% for $Re=10^{-3}$ and from 1.502% to 1.526% for $Re=10^{-4}$. The maximum, mean and centreline percentage errors for each numerical simulation are slightly above 1.5%, which is an acceptable margin of error for three-dimensional cases. The irrelevance of the approximation order for the high-order derivative Neumann-type conditions for the efficiency of the numerical solutions is again exhibited. The five error metrics examined indicate the accuracy of the numerical results produced as satisfactory for both low- and high-order approximations for the second- and third-order derivative Neumann-type conditions. Consequently, in the case of the three-dimensional flow, low-order approximations for the higher-order derivative Neumann-type pressure outlet boundary conditions are sufficient to exceed the limitations of the pseudo-compressibility pressure-velocity coupling formulation of Chorin [23], and the employment of higher-order approximations for the Neumann-type outflow conditions is not necessarily required.

Similar to the previous computational examples, the numerical simulations described by third-order derivative Neumann-type conditions failed to converge to a numerical solution, for $Re\leq 10^{-3}$. In Figure 13, Figure 14, the inappropriateness of the up to fourth-order approximations for the first-derivative Neumann-type pressure boundary conditions at the outlet section, is also exhibited. The examined error metrics for $Re=10^{-1}$, $Re=10^{-2}$, $Re=10^{-3}$ and $Re=10^{-4}$, fall within the range of 95.347% to 98.602%, 95.350% to 98.603%, 95.349% to 98.603% and 95.331% to 98.583%, respectively. The simulations employing the afore-mentioned type of conditions, indicate a deterioration of the flow-field development across the length of the computational domain in very-low Reynolds number (*Re* << 1), incompressible flows governed by the three-dimensional Navier–Stokes equations. As a result, the imposition of low-order derivative Neumann-type boundary conditions for the pressure at the outlet section of the three-dimensional, straight, microfluidic channel flow is not suggested in the context of the pseudo-compressibility method of Chorin [23] to overcome the computational limitations of the formulation in very-low Reynolds number flows, regardless of the order of the approximation. These results come to agree with the remarks inferred from the numerical investigations of the two-dimensional, straight, microfluidic channel flows governed by the Navier–Stokes and the Oseen equations, as mean error percentages ranging from 73% to 85% were observed between the numerical and the analytical solutions of the corresponding two-dimensional computational examples reported in Sec. 3.1.3 and Sec. 3.1.4, respectively.

The computational time required for the numerical simulations prescribing Dirichlet-type and high-order derivative Neumann-type pressure outlet boundary conditions to converge to pseudo steady-state, varies from 6777.41 (min) to 8248.93 (min) for $Re=10^{-1}$, from 1744.15 (min) to 2241.48 (min) for $Re=10^{-2}$, from 2624.47 (min) to 2775.72 (min) for $Re=10^{-3}$ and from 5274.38 (min) to 5643.30 (min) for $Re=10^{-4}$. Though significant, the computational cost is reasonable for the numerical solution of three-dimensional incompressible channel flows in the context of Chorin's [23] pseudo-compressibility method. The number of iterations to achieve convergence to pseudo steady-state, is also reported. For the corresponding range of Reynolds number values, the total number of iterations for the corresponding Reynolds number, bounds between 10064808 to 12251793, 2798055 to 3533614, 4156499 to 4437467 and from 7949808 to 8365782 iterations, respectively. As expected, a lower number of iterations and computational time are required for the numerical simulations employing first-order derivative Neumann-type conditions for the pressure variable at the outlet section of the microfluidic channel, to converge to the designated tolerance for the axial velocity residuals. However, as shown, the imposition of low-order derivative Neumann-type boundary conditions decays the development of the flow across the length of the computational domain, behaviour which was also identified in the case of the two-dimensional computational examples presented in Sections 3.1.3 and 3.1.4.

**Figure 15:**
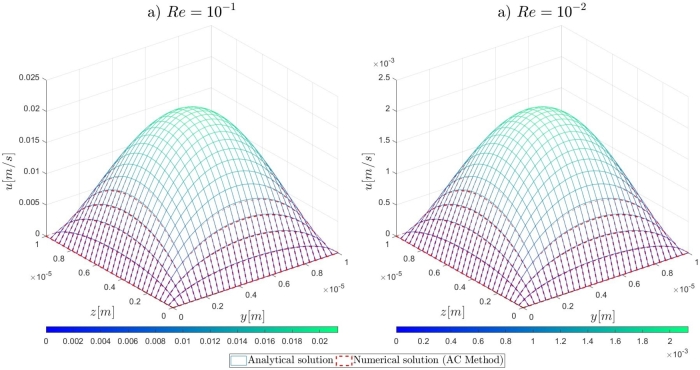
Numerical solution of axial velocity distribution against analytical solution of Eq. (44) at the outlet section and a) *Re* = 10^−1^ and Dirichlet-type condition equal to zero and b) *Re* = 10^−2^ and Dirichlet-type condition equal to *p* = (*ρU*^2^)/2 with a grid size of 30*x*30*x*30.

**Figure 16:**
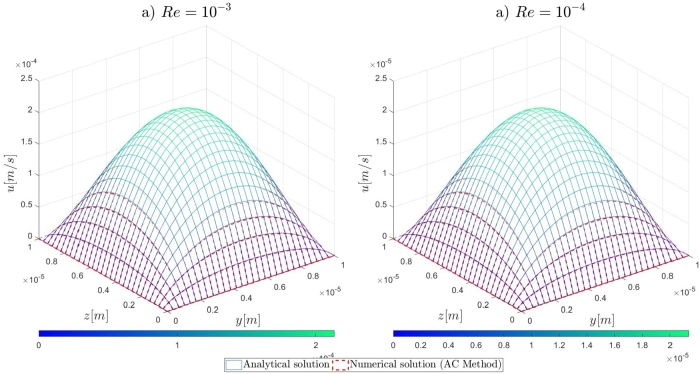
Numerical solution of axial velocity distribution against analytical solution of Eq. (44) at the outlet section and a) *Re* = 10^−3^ and first-order backward approximation *O*(Δ*x*) for the second-order derivative Neumann-type condition and b) *Re* = 10^−4^ and second-order backward approximation *O*(Δ*x*^2^) for the second-order derivative Neumann-type condition with a grid size of 30*x*30*x*30.

A comparison between the estimated numerical solution of the axial velocity component in the context of the pseudo-compressibility method of Chorin [23] and the analytical solution of a steady-state, incompressible, pressure-driven, fully-developed, viscous flow through a rectangular cross-section of Marco and Han [92] at the outlet section of the three-dimensional, microfluidic channel is presented n Figure 15, Figure 16. For the exhibition of the numerically predicted axial velocity surfaces at the outlet section of the three-dimensional, microfluidic channel, the computational results of the numerical simulations employing Dirichlet-type and higher-order derivative Neumann-type conditions have been selected, due to the fact that it was previously shown that they allow for the overcoming of the limitations of Chorin's [23] pseudo-compressibility method at low- and very-low Reynolds numbers (*Re* << 1). The following pressure outflow boundary conditions have been selected: a) a Dirichlet-type condition equal to zero for $Re=10^{-1}$, b) a Dirichlet-type condition equal to the dynamic pressure $p=(\rho U^{2})/2$ for $Re=10^{-2}$, c) a first-order backward approximation O(Δx) for the second-order derivative Neumann-type condition for $Re=10^{-3}$, and d) a second-order backward approximation $O(\Delta x^{2})$ for the second-order derivative Neumann-type condition for $Re=10^{-4}$. The numerical solution of the outlet axial velocity surface for the different types of boundary conditions in the corresponding Reynolds numbers is visualised with solid lines, whilst the analytical solution axial velocity surface with dashed lines. For $Re=10^{-1}$ all five error metrics range from 1.495% to 1.506%, for $Re=10^{-2}$ from 1.506% to 1.517%, for $Re=10^{-3}$ from 1.501% to 1.511% and for $Re=10^{-4}$ the error metrics vary from 1.502% to 1.511%.

It can be clearly seen that for a three-dimensional, straight, microfluidic channel flow, the proposed numerical approach for the mathematical formulation of the pressure outlet boundary condition to overcome the limitation of Chorin's [23] pseudo-compressibility method in very-low Reynolds numbers (*Re* << 1) is appropriate. An overview of the numerical simulations for the three-dimensional, straight, microfluidic channel flow is presented in Table 14, Table 15, Table 16, Table 17.

**Table 14:** Simulation results for three-dimensional, straight, microfluidic channel flow governed by the Navier–Stokes equations against analytical solution of Eq. (44) for different pressure outlet boundary conditions at *Re* = 10^−1^ with a grid size of 30*x*30*x*30.

Reynolds number (*Re* = 10^−1^)				Percentage error against Eq. (44)				
		Simulation metrics		Outlet section		Width middle of outlet section		
Type	Condition	Comp. time (min)	Iterations	Maximum	Mean	Maximum	Mean	Centreline
Dirchlet	0.0	6777.41	10 065 344	1.495%	1.506%	1.495%	1.495%	1.495%
	*ρU*^2^/2	6989.98	10 064 808	1.495%	1.506%	1.495%	1.495%	1.495%
Neumann	*O*(Δ*x*)	1963.46	2 706 742	98.425%	98.602%	98.425%	98.509%	98.425%
(1^*st*^-derivative)	*O*(Δ*x*^2^)	2072.93	2 845 340	96.093%	96.272%	96.093%	96.178%	96.093%
	*O*(Δ*x*^3^)	1928.94	2 874 525	95.664%	95.848%	95.664%	95.751%	95.664%
	*O*(Δ*x*^4^)	1945.44	2 849 595	95.347%	95.536%	95.347%	95.436%	95.348%
Neumann	*O*(Δ*x*)	6813.27	10 155 517	1.527%	1.537%	1.527%	1.526%	1.527%
(2^*nd*^-derivative)	*O*(Δ*x*^2^)	7558.26	10 610 105	1.528%	1.538%	1.528%	1.527%	1.528%
	*O*(Δ*x*^3^)	7460.99	10 610 309	1.528%	1.538%	1.528%	1.527%	1.528%
	*O*(Δ*x*^4^)	7516.88	10 608 956	1.528%	1.538%	1.528%	1.527%	1.528%
Neumann	*O*(Δ*x*)	8248.93	12 251 793	1.532%	1.542%	1.532%	1.531%	1.532%
(3^*rd*^-derivative)	*O*(Δ*x*^2^)	7737.03	11 270 612	1.529%	1.540%	1.529%	1.528%	1.529%
	*O*(Δ*x*^3^)	7399.38	10 997 362	1.529%	1.539%	1.529%	1.528%	1.529%
	*O*(Δ*x*^4^)	7639.29	10 880 724	1.528%	1.539%	1.528%	1.528%	1.528%

**Table 15:** Simulation results for three-dimensional, straight, microfluidic channel flow governed by the Navier–Stokes equations against analytical solution of Eq. (44) for different pressure outlet boundary conditions at *Re* = 10^−2^ with a grid size of 30*x*30*x*30.

Reynolds number (*Re* = 10^−2^)				Percentage error against Eq. (44)				
		Simulation metrics		Outlet section		Width middle of outlet section		
Type	Condition	Comp. time (min)	Iterations	Maximum	Mean	Maximum	Mean	Centreline
Dirchlet	0.0	1842.22	2 915 773	1.507%	1.517%	1.507%	1.506%	1.507%
	*ρU*^2^/2	1829.38	2 915 762	1.507%	1.517%	1.507%	1.506%	1.507%
Neumann	*O*(Δ*x*)	946.61	1 406 199	98.426%	98.603%	98.426%	98.510%	98.426%
(1^*st*^-derivative)	*O*(Δ*x*^2^)	953.73	1 436 782	96.095%	96.273%	96.095%	96.179%	96.095%
	*O*(Δ*x*^3^)	968.43	1 440 820	95.666%	95.849%	95.666%	95.753%	95.666%
	*O*(Δ*x*^4^)	967.29	1 443 556	95.350%	95.537%	95.350%	95.438%	95.350%
Neumann	*O*(Δ*x*)	1744.15	2 798 055	1.514%	1.524%	1.514%	1.513%	1.514%
(2^*nd*^-derivative)	*O*(Δ*x*^2^)	1882.54	2 984 454	1.514%	1.524%	1.514%	1.513%	1.514%
	*O*(Δ*x*^3^)	1871.86	2 985 082	1.514%	1.524%	1.514%	1.513%	1.514%
	*O*(Δ*x*^4^)	1895.09	2 984 919	1.514%	1.524%	1.514%	1.513%	1.514%
Neumann	*O*(Δ*x*)	2241.48	3 533 614	1.515%	1.525%	1.515%	1.514%	1.515%
(3^*rd*^-derivative)	*O*(Δ*x*^2^)	2033.57	3 204 626	1.515%	1.525%	1.515%	1.514%	1.515%
	*O*(Δ*x*^3^)	1979.93	3 114 580	1.515%	1.525%	1.515%	1.514%	1.515%
	*O*(Δ*x*^4^)	1945.80	3 076 672	1.515%	1.525%	1.515%	1.514%	1.515%

**Table 16:** Simulation results for three-dimensional, straight, microfluidic channel flow governed by the Navier–Stokes equations against analytical solution of Eq. (44) for different pressure outlet boundary conditions at *Re* = 10^−3^ with a grid size of 30*x*30*x*30.

Reynolds number (*Re* = 10^−3^)				Percentage error against Eq. (44)				
		Simulation metrics		Outlet section		Width middle of outlet section		
Type	Condition	Comp. time (min)	Iterations	Maximum	Mean	Maximum	Mean	Centreline
Dirchlet	0.0	2775.72	4 437 467	1.514%	1.525%	1.514%	1.514%	1.514%
	*ρU*^2^/2	2772.48	4 437 467	1.514%	1.525%	1.514%	1.514%	1.514%
Neumann	*O*(Δ*x*)	2252.79	3 332 530	98.426%	98.603%	98.426%	98.510%	98.426%
(1^*st*^-derivative)	*O*(Δ*x*^2^)	2258.40	3 390 610	96.094%	96.273%	96.095%	96.179%	96.095%
	*O*(Δ*x*^3^)	2108.44	3 144 939	95.666%	95.849%	95.666%	95.752%	95.666%
	*O*(Δ*x*^4^)	2116.24	3 140 136	95.349%	95.537%	95.349%	95.438%	95.350%
Neumann	*O*(Δ*x*)	2624.47	4 156 499	1.502%	1.511%	1.502%	1.501%	1.502%
(2^*nd*^-derivative)	*O*(Δ*x*^2^)	2735.36	4 301 337	1.503%	1.512%	1.503%	1.501%	1.503%
	*O*(Δ*x*^3^)	2711.22	4 301 902	1.503%	1.512%	1.503%	1.501%	1.503%
	*O*(Δ*x*^4^)	2702.48	4 301 568	1.503%	1.512%	1.503%	1.501%	1.503%

**Table 17:** Simulation results for three-dimensional, straight, microfluidic channel flow governed by the Navier–Stokes equations against analytical solution of Eq. (44) for different pressure outlet boundary conditions at *Re* = 10^−4^ with a grid size of 30*x*30*x*30.

Reynolds number (*Re* = 10^−4^)				Percentage error against Eq. (44)				
		Simulation metrics		Outlet section		Width middle of outlet section		
Type	Condition	Comp. time (min)	Iterations	Maximum	Mean	Maximum	Mean	Centreline
Dirchlet	0.0	5278.61	7 949 808	1.507%	1.514%	1.507%	1.504%	1.507%
	*ρU*^2^/2	5274.38	7 949 808	1.507%	1.514%	1.507%	1.504%	1.507%
Neumann	*O*(Δ*x*)	4498.25	6 710 394	98.403%	98.583%	98.403%	98.489%	98.403%
(1^*st*^-derivative)	*O*(Δ*x*^2^)	4525.18	6 738 286	96.075%	96.257%	96.075%	96.161%	96.075%
	*O*(Δ*x*^3^)	4730.32	6 777 834	95.647%	95.833%	95.647%	95.735%	95.648%
	*O*(Δ*x*^4^)	4547.37	6 772 497	95.331%	95.522%	95.332%	95.421%	95.332%
Neumann	*O*(Δ*x*)	5643.30	8 365 782	1.513%	1.526%	1.513%	1.514%	1.513%
(2^*nd*^-derivative)	*O*(Δ*x*^2^)	5487.33	8 199 666	1.504%	1.511%	1.504%	1.502%	1.504%
	*O*(Δ*x*^3^)	5517.21	8 201 311	1.504%	1.511%	1.504%	1.502%	1.504%
	*O*(Δ*x*^4^)	5494.19	8 200 462	1.504%	1.511%	1.504%	1.502%	1.504%

## 4 Conclusions

In the present work, a numerical approach has been proposed to overcome a long-standing challenge in the field of applied mathematical modelling for the limitations of the pseudo-compressibility method of Chorin [23] at very-low Reynolds number steady-state, viscous-dominant, incompressible flows (*Re* << 1). The basis of the approach is relying on the treatment of the pressure outlet boundary condition with higher-order Neumann-type pressure boundary conditions along with their up to fourth-order numerical treatment which have been proposed in Section 2. The numerical approach proposed for the treatment of the pressure outlet boundary conditions employed the standard explicit finite difference method for the spatial and an explicit third-order Runge–Kutta pseudo-time integration scheme for the pseudo-temporal discretisation. The benchmark test problems have been considered for two-dimensional, straight, microfluidic flows in a channel governed by the Navier–Stokes and Oseen equations separately, and three-dimensional, straight, microfluidic flows governed by the Navier–Stokes equations in cartesian coordinates. The application of the pseudo-compressibility method of Chorin [23] for the mathematical modelling and the numerical solution of the low and very-low Reynolds number Oseen flow (*Re* << 1) was first presented in this study. The numerical solution required for the perturbed continuity equation, in addition to the absence of a Poisson equation for the determination of the pressure-field, dictate the need for the special treatment of the pressure boundary conditions. Regarding the treatment of the pressure variable at the outlet section of the examined channels, Dirichlet-type conditions are also employed, and up to fourth-order approximations for the first-, second- and third-order derivative Neumann-type conditions were constructed. The impact of 14 different mathematical formulations for the pressure outlet boundary condition on the fluid flow development and deterioration across the length of the computational domain was investigated at 4 different Reynolds numbers, ranging from $10^{-1}$ to $10^{-4}$. For validation purposes, the simulation results were compared against simplified analytical solutions available in the literature for two- and three-dimensional microfluidic channel flows.

The obtained results indicate that the employment of Dirichlet-type and high-order derivative Neumann-type conditions for the pressure at the outlet section of the channels is appropriate for the flow-field development. In contrast, the first-order derivative Neumann-type pressure outlet boundary conditions leads to a deterioration of the fluid flow-field and yielded non-physical results. The imposition of the currently proposed model to overcome the computational limitations of the pseudo-compressibility method for the investigated Reynolds number range, resulted in percentage errors approximately below 1% for each numerical simulation performed. Conversely, low-order derivative Neumann-type pressure outlet boundary conditions resulted in numerical solutions with percentage errors ranging from approximately 73% to 85% for the investigated two-dimensional, straight, microfluidic channel flows and above 95% for the three-dimensional, microfluidic channel flows. Another conclusion derived is the sufficiency of low-order approximations for the second- and third-derivative Neumann-type conditions, comparing to higher-order approximations. Particularly, the present study indicates that the computational focus should be adverted to the order of the employed derivatives for the Neumann-type boundary conditions, instead of the order of the finite difference approximation for the corresponding derivative. For instance, it is shown that the imposition of first-order approximations for the second-order derivative Neumann-type conditions allows for the accurate flow-field development, whereas the employment of fourth-order approximations for the first-order derivative Neumann-type pressure boundary condition results in deterioration of the fluid flow field. The limitations of the pseudo-compressibility method of Chorin [23] for viscous-dominant flows (*Re* << 1) was highlighted in the literature (see Section 1). Therefore, through a special mathematical treatment of the outlet boundary condition for the pressure variable, the numerical approach proposed in this work successfully overcomes the computational limitations of the pseudo-compressibility method with the use of finite difference method (FDM) for low- and very-low Reynolds number (*Re* << 1) incompressible flows. Particularly, the mathematical treatment for the pressure outlet boundary conditions proposed in the present study allows for the employment of Chorin's [23] hyperbolic-type pseudo-compressibility method in a wider range of engineering applications beyond its previously known constraints at very-low Reynolds number flows (*Re* << 1).

However, the proposed numerical approach presents specific limitations. In this work, the formulation of the employed discretisation schemes can only be applied in structured meshes. Therefore, the computational examples investigated are based on geometric configurations for the two- and three-dimensional, straight, microfluidic channels in Cartesian coordinates. The work of Fornberg [75] adopted in the present work focuses on the generation of finite difference formulations on arbitrarily spaced grids. Future studies may include the extension of the proposed numerical approach to account for configurations characterised by curvilinear coordinate systems through the employment of Jacobian transformations, allowing for the application of the introduced numerical approach in any type of coordinate system and regardless of the investigated geometric configuration. The contribution of the present investigation on overcoming the computational limitations of the pseudo-compressibility method of Chorin [23] in very-low Reynolds number flows using high-order derivative pressure outflow Neumann-type boundary conditions is independent from the shape of the computational domain. The shape of the domain especially in the case of an unstructured mesh can be taken into consideration through the transformed discretisation schemes employed for the solution of the fluid flow governing equations and the boundary conditions. In addition, the currently proposed model focuses on pressure-driven flows where the imposition of a high-order pressure outlet boundary condition is required for the accurate flow-field development and the absence of flow deterioration phenomena. Lastly, in the governing equations of the present study, no external forces are assumed to be acting on the fluid body. Future studies may investigate the influence of the inclusion of source terms in the governing equations describing the microfluidic channel flows, to further comprehend the importance of employing high-order pressure boundary conditions at the outlet section of the investigated geometric configurations. For instance, in the case of magneto- and ferrohydrodynamic (MHD-FHD) fluid flow problems, the inclusion of additional source terms in the governing equations is essential. Apart from the expected modifications regarding the inclusion of the magnetization source terms in the governing equations, further adjustments are expected for the systematic prediction of the behaviour of the AC parameter *β*. In this way, the model proposed here may constitute the basis to overcome the computational limitations of the pseudo-compressibility method of Chorin [23] in very-low Reynolds number, incompressible flows for a broad range of engineering flows.

## CRediT authorship contribution statement

**Nikos Monokrousos:** Writing – review & editing, Writing – original draft, Visualization, Validation, Software, Methodology, Investigation, Formal analysis, Data curation, Conceptualization. **László Könözsy:** Writing – review & editing, Validation, Supervision, Resources, Project administration, Methodology, Investigation, Funding acquisition, Formal analysis, Conceptualization. **Vassilios Pachidis:** Writing – review & editing, Supervision, Resources, Project administration, Investigation, Funding acquisition, Formal analysis, Conceptualization. **Ernesto Sozio:** Writing – review & editing, Validation, Supervision, Resources, Project administration, Funding acquisition, Formal analysis, Conceptualization. **Federico Rossi:** Writing – review & editing, Validation, Supervision, Resources, Project administration, Funding acquisition, Formal analysis, Conceptualization.

## Declaration of Competing Interest

The authors declare that they have no known competing financial interests or personal relationships that could have appeared to influence the work reported in this paper.
